# Preclinical evidence and potential mechanisms of tanshinone ⅡA on cognitive function in animal models of Alzheimer’s disease: a systematic review and meta-analysis

**DOI:** 10.3389/fphar.2025.1603861

**Published:** 2025-07-11

**Authors:** Yuanhang Rong, Qinqing Li, Yuzhong Du, Wenting Wang, Wenna Su, Junlong Zhang, Wenbin He

**Affiliations:** ^1^ College of Traditional Chinese Medicine, Shandong University of Traditional Chinese Medicine, Jinan, Shandong, China; ^2^ Shanxi Key Laboratory of Chinese Medicine Encephalopathy, Shanxi University of Chinese Medicine, Jinzhong, Shanxi, China; ^3^ National International Joint Research Center for Molecular Traditional Chinese Medicine, Shanxi University of Chinese Medicine, Jinzhong, Shanxi, China; ^4^ School of Pharmacy, Shanxi Medical University, Jinzhong, Shanxi, China

**Keywords:** Alzheimer’s disease, animal models, cognitive function, mechanisms, systematic review and meta-analysis, Tanshinone ⅡA

## Abstract

**Background:**

Tanshinone ⅡA (Tan ⅡA) is a monomer extracted from *Salvia miltiorrhiza* Bunge. Animal studies have demonstrated its potential in providing cognitive protection in Alzheimer’s disease (AD), but the overall effects remain inconclusive, and its multiple mechanisms have not been systematically summarized.

**Objective:**

This systematic review and meta-analysis (SR/MA) aimed to evaluate the overall effects of Tan ⅡA on cognitive function in AD animal models and to summarize the mechanisms.

**Methods:**

Seven databases (PubMed, Embase, Web of Science, China National Knowledge Infrastructure, Chinese Biological Medical Disc, Chongqing VIP, and Wanfang databases) and grey literature were retrieved. Risk of bias was evaluated following the Systematic Review Center for Laboratory Animal Experiments. The mean difference (MD) or standard mean difference (SMD) with 95% confidence intervals (CIs) were used to evaluate the effect of Tan ⅡA on cognitive function, neuropathology, neuroinflammation, oxidative stress, apoptosis, and neural/synaptic plasticity, with *P* < 0.05 considered a significant difference. The effect and potential mechanisms of Tan ⅡA were demonstrated by performing multiple subgroup analyses.

**Results:**

Nineteen studies involving 581 AD animals were identified. The included studies showed satisfactory reporting quality but had certain risks of bias in methodology. Tan ⅡA ameliorated cognitive deficits, evidenced by reducing escape latency (MD = −17.94 s; 95% CI: −22.92 to −12.96) and increasing time spent in the target quadrant (MD = 10.69 s; 95% CI: 7.32–14.07). It attenuated neuropathological damage by reducing amyloid-β (Aβ) plaques in thioflavine S staining (SMD = −3.46; 95% CI: −5.65 to −1.26) and increasing neuronal density in Nissl staining (SMD = 2.82; 95% CI: 2.11–3.52) and NeuN staining (SMD = 2.89; 95% CI: 1.71–4.08). Tan ⅡA also demonstrated anti-inflammatory effects through downregulation of pro-inflammatory cytokines [tumor necrosis factor-alpha (TNF-α), interleukin-1 beta (IL-1β), and interleukin-6 (IL-6)] and antioxidant stress properties by increasing superoxide dismutase (SOD) and glutathione peroxidase (GSH-Px) levels while reducing reactive oxygen species (ROS) and malondialdehyde (MDA) levels. Additionally, it exhibited antiapoptotic effects by increasing the B-cell lymphoma-2/Bcl-2-associated X protein (Bcl-2/Bax) ratio and decreasing Caspase-3 expression. Moreover, treatment improved neuronal/synaptic plasticity by upregulating postsynaptic density-95 (PSD-95) and brain-derived neurotrophic factor (BDNF) levels.

**Conclusion:**

Tan ⅡA could improve cognitive function and neuropathology through multiple mechanisms. This suggests that Tan IIA may serve as a viable candidate for the development of therapeutic strategies for AD.

**Systematic review registration:**

https://www.crd.york.ac.uk/PROSPERO/view/CRD42024588415.

## Introduction

Alzheimer’s disease (AD), which is a progressive and irreversible neurodegenerative disorder characterized by multi-domain cognitive decline, represents the most prevalent form of dementia worldwide (Scheltens et al., 2021). A person with AD may suffer from cognitive dysfunction, memory decline, aphasia, and apraxia; personality and behavior changes; and even gradually lose the self-care ability, eventually becoming completely reliant on others in daily life, which causes a huge burden on the family and society. The most recent data show that approximately 55 million people worldwide are suffering from dementia, and it is estimated that it will reach 140 million by 2050 (Huang et al., 2023). Accounting for 60%–70% of dementia cases, AD contributes most to the increase in the number of people with dementia, and is considered to be an expensive, lethal, and burdening disease (Scheltens et al., 2021; Gustavsson et al., 2023). Multiple hypotheses have been proposed to explain the pathogenesis of AD, including the formation of extracellular amyloid-β (Aβ) plaques and intracellular Tau-based neurofibrillary tangles (NFTs), which are considered the primary pathology of AD (Zhai et al., 2024). In addition, loss of cholinergic neurons (Moreira et al., 2022), neuroinflammation (Saha et al., 2021), oxidative stress (Cecerska-Heryć et al., 2022), and synaptic dysfunction (Driscoll et al., 2024) are also considered to be involved in AD. In conclusion, AD presents complex and diverse pathological changes. Due to the incomplete understanding of its precise mechanisms, there is currently no effective curative treatment available for AD (Walsh et al., 2024). Despite the remarkable progress in the field of pharmacological AD therapies in the past two decades, the results of phase Ⅲ clinical trials of novel drugs are controversial, with significant adverse events (Walsh et al., 2021; Cummings et al., 2024). Due to the intricate neuropathologic processes of AD, single-target and single-pathway drugs can hardly prevent or reverse AD progression. Therapeutic strategies focusing on multi-target and multi-mechanism interventions are expected to become the mainstream.

**FIGURE 1:**
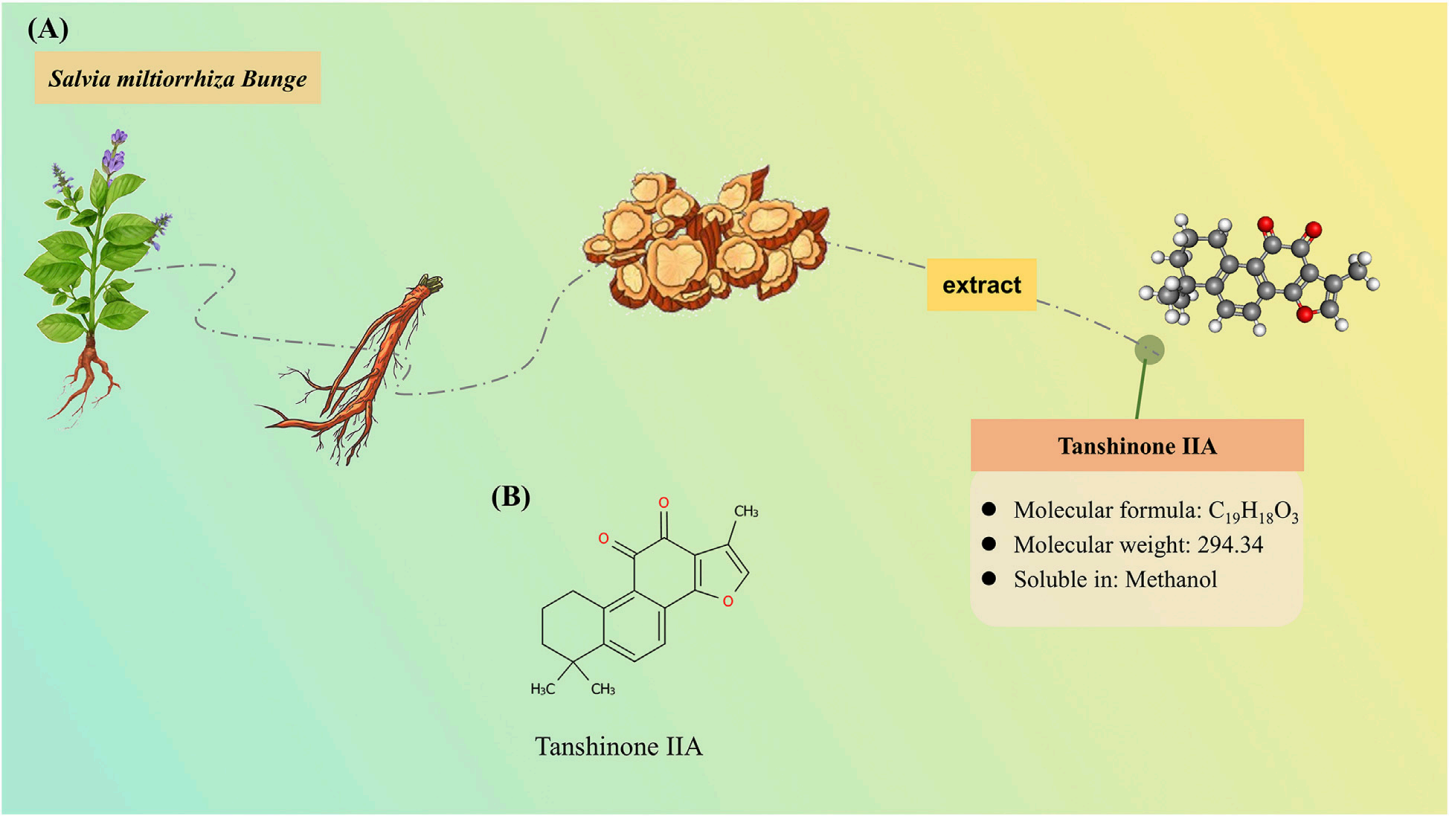
Resource and characteristics of tanshinone IIA (Tan IIA). **(A)** Tan IIA, as a lipophilic compound, is derived from the rhizomes of the herbaceous plant *Salvia miltiorrhiza Bunge*. **(B)** Chemical structure of Tan IIA.

In recent decades, traditional Chinese medicine (TCM) has emerged as a promising therapeutic strategy and offers a holistic approach to both prevention and management of AD. The active components of TCM serve as the material basis for its therapeutic effects. Tanshinone ⅡA (Tan ⅡA) is a lipophilic diterpene isolated from the rhizome of traditional Chinese herb *Salvia miltiorrhiza* Bunge. Tan ⅡA exhibits promising neuroprotective effects on many neurological disorders, including stroke (Arefnezhad et al., 2024), AD (Fang et al., 2024), Parkinson’s disease (Zhang et al., 2015), and epilepsy (Ma et al., 2024), with diverse pharmacological properties, such as regulating apoptosis, inflammation, and oxidative stress (Zhong et al., 2021). The resource and characteristics of Tan ⅡA are illustrated in Figure 1.

A growing body of preclinical animal experimental studies has been carried out to investigate the therapeutic potential of Tan ⅡA for AD. However, the overall efficacy remains inconclusive due to limited sample sizes and insufficient statistical power across individual studies. There is a shortage of meta-analysis on this topic. Network pharmacology (Xiang et al., 2024a) indicates that Tan ⅡA ameliorates AD cognitive function through multiple targets and signaling pathways. However, existing research has predominantly focused on isolated pathways or a narrow range of efficacy markers, leaving the complex and multifaceted mechanisms of Tan ⅡA in AD treatment incompletely characterized. Based on this, the systematic review and meta-analytic approaches are used in this study to assess the cognitive protective benefits of Tan ⅡA in AD models and integrate findings from mechanistic studies, thereby providing preclinical evidence for its clinical application.

The present systematic review and meta-analysis (SR/MA) was designed to address the following objectives:

• evaluate the efficacy of Tan ⅡA on cognitive function in AD animal models;
• summarize the possible mechanisms of Tan ⅡA in the treatment of AD;
• investigate the factors influencing the efficacy of Tan ⅡA in AD animal models.

## Methods

This systematic review was performed in accordance with the Preferred Reporting Items for Systematic Reviews and Meta-Analyses (PRISMA) guidelines (Moher et al., 2010), registered on the International Prospective Register of Systematic Reviews (PROSPERO) platform, and the protocol was accessed at https://www.crd.york.ac.uk/PROSPERO/view/CRD42024588415.

### Search strategy

Seven databases (PubMed, Embase, Web of Science, China National Knowledge Infrastructure, Chinese Biological Medical Disc, Chongqing VIP, and Wanfang databases) were searched from inception to May 2025, with the last search on 5 May 2025. The search terms and their extensions, including “tanshinone ⅡA,” “Alzheimer* disease,” “Alzheimer type dementia,” “senile dementia,” “animal experiment*,” and “*in vivo*,” were connected using Boolean operators “AND” or “OR” to retrieve compliant publications as comprehensively and accurately as possible. The search strategy was customized to suit the specific requirements of databases. The search strategies in seven databases are provided in Supplementary Material S1 and S2. A manual screening of reference lists from included studies was conducted to identify potentially eligible publications. In addition, searches of grey literature databases (OpenGrey, bioRxiv, and Google Scholar) and identification of industry-sponsored studies were also conducted. All records were deduplicated using EndNote X9, followed by Microsoft Excel.

### Eligibility criteria

The eligibility criteria were established under the guidance of the PICOS framework. P (population): rat or mouse models with AD; I (intervention): Tan ⅡA; C (control): vehicle-treated animals after modeling or transgenic animal model of AD undergoing no treatment at all; O (outcomes): cognitive function assessed through the Morris water maze (MWM) test and pathological changes in the brain, neuroinflammation, oxidative stress, apoptosis, and neural and synaptic plasticity; and S (study design): preclinical controlled studies.

Inclusion criteria were as follows:

• preclinical experimental studies in rats or mice, with no restriction on strain, species, sex, and model;
• Tan ⅡA, with no restriction on the preparation, dosage, administration route, and treatment duration;
• at least one of the control groups was the AD model group, without treatment or given equal volume of vehicle;
• outcome indicators: a. Primary outcome measures: cognitive function tested through MWM (escape latency and time spent in the target quadrant). b. Secondary outcome measures: pathological changes in the brain (Aβ plaque and neuronal damage), inflammation-related factors [tumor necrosis factor-alpha (TNF-α), interleukin-1 beta (IL-1β), and interleukin-6 (IL-6)], oxidative stress-related indicators [superoxide dismutase (SOD), glutathione peroxidase (GSH-Px), reactive oxygen species (ROS), and malondialdehyde (MDA)], apoptosis markers [caspase-3 and B-cell lymphoma-2/Bcl-2-associated X protein (Bcl-2/Bax)], and neural and synaptic plasticity-related proteins [postsynaptic density-95 (PSD-95) and brain-derived neurotrophic factor (BDNF)].

Exclusion criteria were as follows:

• combined with other therapies in addition to Tan ⅡA;
• full text unavailable, incomplete data, or with obvious errors;
• duplicate publication of the same experimental data.

### Study selection and data extraction

A two-step screening process was used for study selection. In the first step, two research workers (YR and YD) browsed titles and abstracts back-to-back to select initially qualified studies. The studies that were not included were classified according to the exclusion criteria. The second step was a full-text review by the same two research workers independently against predetermined inclusion criteria, followed by a double-checking. Any disagreements were resolved through consultation with a third reviewer (JZ). If two articles with identical content were published in different languages, only one was retained. If two articles stemmed from the same study and utilized the same batch of animals but reported different outcomes, the one containing more information was reserved and absorbed outcomes of another.

Before data extraction, a data extraction form was designed and subsequently refined after being piloted on three studies. Two research workers (YR and WW) independently extracted data for relevant outcomes in accordance with the updated form. Complete data were obtained by emailing to the author in case where there were unreported or unclear data. The key information extracted encompassed author identification (author ID and date), animal characteristics (species, strain, age, weight, model, and number of each group), intervention (administration route, dose, and treatment duration), and outcomes. The inter-rater reliability was assessed using Cohen’s kappa. For outcome indicators measured for several consecutive days, only the value of the last day was taken. Original values with actual units were recorded truthfully and then consistently converted across studies for comparability. For continuous outcomes, the mean value and standard deviation (SD) were extracted, and data were converted using the formula SD = √ n × SEM, when standard error of the mean (SEM) was given (where n represents the number of animals). WebPlotDigitizer 4.5 was used when data were presented graphically. In the presence of multiple-dose intervention subgroups, the mean values and SDs were combined into a single group using the following formula according to the Cochrane Handbook for Systematic Reviews of Interventions (CHSRI):

Sample size: N_1_ + N_2_

$$\text{Mean}:\left(N_{1}M_{1}+N_{2}M_{2}\right)/\left(N_{2}M_{2}\right),$$

$$\text{SD}:\sqrt{\frac{\left(\left(N_{1}-1\right){SD}_{1}^{2}+\left(N_{2}-1\right){SD}_{2}^{2}+\frac{\left(N_{1}N_{2}\right)}{\left(N_{1}+N_{2}\right)\left(M_{1}^{2}+M_{2}^{2}-2M_{1}M_{2}\right)}\right)}{\left(N_{1}+N_{2}-1\right)}.}$$

### Quality assessment and risk of bias

Two research workers (YR and WS) independently used a 10-item scoring checklist adapted from the study by Liu et al. (2024a) to assess the reporting quality of included studies. For an individual study, each parameter was rated as “Yes,” “No,” or “Partly.” Studies awarded “Yes” in more than 70% of parameters were regarded as high quality, whereas those awarded “Yes” in less than 50% were considered as low quality, and those awarded “Yes” in 50%–70% were considered as medium quality. The risk of bias was evaluated by two independent research workers (YR and WS) using the SYRCLE’s RoB tool (Hooijmans et al., 2014), which consists of 10 items covering the following six aspects: selection bias, performance bias, detection bias, attrition bias, reporting bias, and other biases. The categories were evaluated as “Low,” “High,” and “Unclear,” which represented low risk, high risk, and unclear risk of bias, respectively. The inter-rater reliability was assessed using Cohen’s kappa. Any discrepancies between the research workers were resolved by consulting a third research worker (QL).

### Data analysis

Review manager 5.3 and Stata 15.0 were used for data analysis. Given that all the outcomes were predefined as continuous variables, mean differences (MDs) or standardized mean differences (SMDs) and their corresponding 95% confidence intervals (95% CIs) were calculated as effect sizes, with the cutoff for statistical significance set at *P* < 0.05. On the one hand, when natural units were used to measure the outcome indicator, MD should be used. On the other hand, when the method or unit of measurement showed cleavage among studies, SMD was a preferred option. Random-effects models were employed in this meta-analysis and pooled analyses were visualized with forest plots. The chi-square statistic and the Higgins index (I^2^) were used to quantify heterogeneity among studies, and results with I^2^ >50% were considered substantial, which implied the existence of considerable heterogeneity. If meta-analysis was not feasible, a qualitative synthesis was adopted to report the data.

### Subgroup analyses and meta-regression

To investigate the factors influencing the efficacy of Tan ⅡA in AD animal models and explore the sources of heterogeneity, subgroup analyses were conducted based on animal characteristics (species and model) and intervention characteristics (administration route, dose, and treatment duration). For studies with multiple dose groups, the average dose was used for classification. Furthermore, meta-regression was conducted using Stata 15.0 software to assess the potential influence of covariates on heterogeneity when an adequate number of studies were available.

### Publication bias

The funnel plot and Egger’s test complemented each other in assessing the publication bias of the included studies. When there was a sufficient quantity of studies related to the outcome, funnel plots were constructed to visually assess the potential presence of publication bias. A uniform distribution of scatter points around the combined effect size indicated the absence of publication bias. Egger’s test provided a quantitative measure of publication bias, with *P* < 0.05 indicating the presence of such bias.

### Sensitivity analysis

Sensitivity analysis using the “leave-one-out” method was conducted to evaluate the robustness of the meta-analysis results. In the circumstance where there was minimal influence on the overall outcome after removal, it was reasonable to believe that the results of the meta-analysis were reliable.

## Results

### Study selection

**FIGURE 2:**
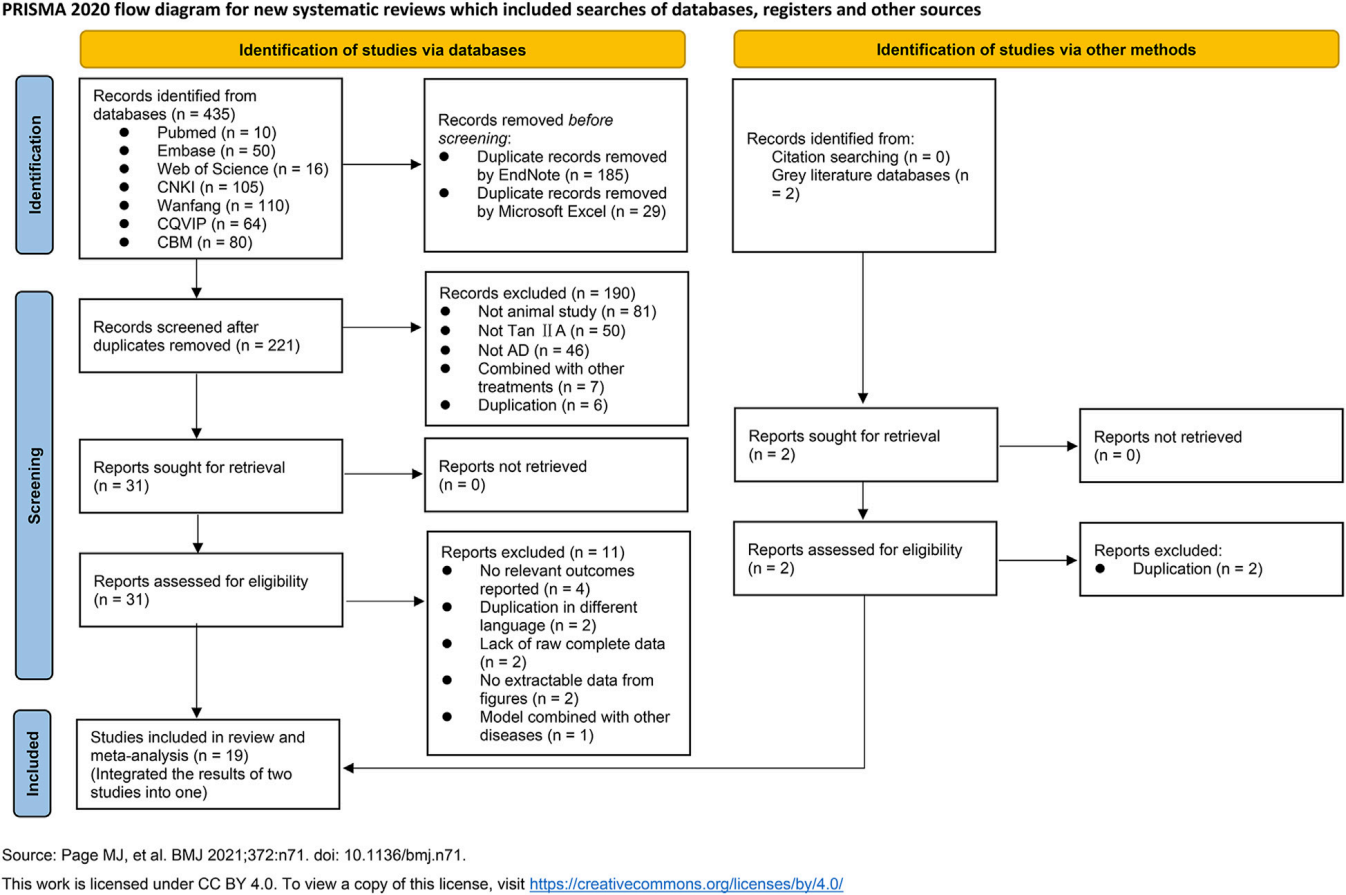
PRISMA flowchart for literature screening.

A total of 435 relevant studies were identified from seven typical databases, including 76 in English and 359 in Chinese. After removing duplicates using EndNote software and Microsoft Excel, 221 records remained; two reviewers then screened the titles and abstracts and excluded 190 irrelevant studies. Full texts of 31 studies were further assessed for eligibility, and 11 studies were excluded. Out of them, four reported irrelevant outcomes, two were content duplications in different languages, four lacked raw complete data or were failed to be extracted data from figures, and one used animal model of AD combined with another diseases. Given that two studies used the same batch of animals and experimental methods but reported different outcomes, likely arising from the same project, we integrated their outcomes into one study. Two records were retrieved from citations and grey literature databases, but both were duplicates already included in the previously searched typical databases. Ultimately, a total of 19 studies were included for qualitative or quantitative synthesis (Figure 2).

### Study characteristics

Nineteen studies were included involving 771 animals (581 AD and 190 normal controls). Among the AD animals, 190 were AD controls, whereas 391 were treated with Tan ⅡA. Positive-drug control groups were excluded from data analysis. Except for one study (Jiang et al., 2010) with an equal ratio of male and female rodents, all other studies used male rodents, subordinating to 333 mice (57%) and 248 rats (43%). In these 19 studies, Sprague–Dawley (SD) rats were the most common species (240 in 8 studies, 41%), followed by B6C3-Tg mice (195 in 7 studies, 34%). Kunming (40 in 1 study, 7%), C57BL/6J (18 in 1 study, 3%), ICR (48 in 1 study, 8%), and Swiss albino (40 in 1 study, 7%) mice were also used. There were four approaches to developing AD models: injection of Aβ (eight studies), transgenic AD (APP/PS1) (seven studies), streptozotocin (STZ) (three studies), and lipopolysaccharide (LPS) (one study).

Tan ⅡA was either commercially sourced from biotechnology companies or extracted in laboratory by research workers, with administration routes including both intraperitoneal injection and oral gavage. The dosages used in these 19 studies ranged from 1 mg/kg to 100 mg/kg. Thirteen studies conducted dose–control experiments on Tan ⅡA, with two or three dose groups set up. The remaining six studies set one dose group. Cohen’s kappa between YR and WW was 0.92. Further details are presented in Table 1.

**TABLE 1:** Study characteristics of including studies (n = 19).

Study ID	Species	Strain	Sex	Age/weight	Model	NC	NE	Administration route	Dose (mg/kg)	Treatment duration (days)	Related outcome	
Ding et al. (2020)	Mice	B6C3-Tg	M	6 months/NA	APP/PS1	10	20	Intraperitoneal injection	5; 20	30	Ethology	MWM
											Pathological changes	Aβ plaques (IHC and Th-S), neuron (NeuN)
											Neuroinflammation	TNF-α, IL-1β, and IL-6
											Neural and synaptic plasticity	PSD-95
											Signaling pathways	RAGE/NF-κB
											Other	Number of microglia and astrocytes, IBA-1, and GFAP (activation of glial cells)
Fang et al. (2021)	Mice	ICR	M	7 weeks/18∼22 g	LPS	12	36	Intraperitoneal injection	1; 5; 10	49	Ethology	MWM
											Pathological changes	Neuron (HE)
											Neuroinflammation	TNF-α and IL-6
											Neural and synaptic plasticity	BDNF
											Signaling pathways	NF-κB and PI3K/Akt
											Other	IBA-1 and GFAP (activation of glial cells) ACh and AChE (cholinergic system)
He et al. (2020a)	Mice	KM	M	7∼8 weeks/30∼35 g	STZ	10	30	Oral gavage	20; 40; 80	30	Ethology	MWM
											Pathological changes	Neuron (Nissl)
											Apoptosis	Caspase-3 and Bcl-2/Bax
											Signaling pathways	PERK-eIF2α, ATF6, CHOP, JNK, and Caspase
He et al. (2020b)	Mice	B6C3-Tg	M	6 months/25∼30 g	APP/PS1	7	14	Intraperitoneal injection	10; 30	28	Ethology	MWM
											Pathological changes	Aβ plaques (IHC and Th-S)
											Apoptosis	Caspase-3 and Bcl-2/Bax
											Signaling pathways	PERK-eIF2α, ATF6, CHOP, JNK, and Caspase
Jiang et al. (2010)	Rats	SD	M&F	8∼12 weeks/200∼250 g	Aβ_1-42_	15	15	Oral gavage	50	15	Ethology	MWM
											Oxidative stress	ROS
Jiang et al. (2014)	Rats	SD	M	8∼12 weeks/200∼250 g	Aβ_1-42_	15	15	Oral gavage	50	15	Ethology	MWM
											Signaling pathways	NF-κB
Li et al. (2015)	Rats	SD	M	NA/220∼280 g	Aβ_1-42_	10	10	Intraperitoneal injection	8	30	Pathological changes	Aβ plaques (IHC) and neuron (Nissl and NeuN)
											Signaling pathways	NF-κB
											Other	GFAP (activation of glial cells)
Li et al. (2016)	Mice	B6C3-Tg	M	6 months/NA	APP/PS1	10	30	Intraperitoneal injection	25; 50; 100	30	Ethology	MWM
											Neural and synaptic plasticity	BDNF
											Signaling pathways	BDNF-TrkB
											Other	p-Tau/t-Tau, APP mRNA, Aβ, and electrophysiology
											Neuroinflammation	TNF-α and IL-1β
											Other	IBA-1 (activation of glial cells)
Lin et al. (2019)	Rats	SD	M	4 months/NA	Aβ1-42	10	30	Oral gavage	20; 40; 80	28	Ethology	MWM
											Pathological changes	Neuron (Nissl)
											Apoptosis	Caspase-3
											Signaling pathways	ERK1/2
											Other	p-Tau/t-Tau
Liu et al. (2016)	Mice	Swiss albino	M	4 months/30∼35 g	STZ	10	30	Intraperitoneal injection	20; 40; 80	28	Ethology	MWM
											Pathological changes	Neuron (Nissl)
											Oxidative stress	SOD, GSH-Px, and MDA
											Signaling pathways	MAPK
											Other	AChE (cholinergic system)
Liu et al. (2024b)	Mice	B6C3-Tg	M	2 months/NA	APP/PS1	10	20	Oral gavage	10; 20	56	Ethology	MWM
											Oxidative stress	SOD, GSH-Px, and MDA
											Apoptosis	Caspase-3 and Bcl-2/Bax
											Neural and synaptic plasticity	BDNF and PSD-95
											Signaling pathways	PI3K/Akt
											Other	Ach, AchE, and ChAT (cholinergic system)
Lu et al. (2016)	Rats	SD	M	NA/220∼280 g	Aβ_1-42_	10	10	Intraperitoneal injection	8	30	Neuroinflammation	IL-1β
											Pathological changes	Neuron (HE)
											Other	GFAP (activation of glial cells)
Luo (2014)	Mice	B6C3-Tg	M	12 months/28∼31 g	APP/PS1	7	7	Intraperitoneal injection	4	60	Pathological changes	Aβ plaques (IHC) and neuron (Nissl)
											Other	C3 and C1q (complement)
											Pathological changes	Aβ plaques (Th-S)
											Oxidative stress	SOD, MDA, and ROS
											Neural and synaptic plasticity	BDNF and PSD-95
											Signaling pathways	GLUT1/LRP1
											Other	ChAT and AChE (cholinergic system)
Peng et al. (2022)	Mice	B6C3-Tg	M	5 months/NA	APP/PS1	10	20	Intraperitoneal injection	15; 30	28	Ethology	MWM
											Pathological changes	Neuron (Nissl and NeuN)
											Oxidative stress	SOD, GSH-Px, and MDA
											Apoptosis	Caspase-3 and Bcl-2/Bax
											Signaling pathways	PI3K/Akt
											Other	p-Tau/t-Tau AChE and ChAT (cholinergic system)
Ren et al. (2024)	Rats	SD	M	NA/200∼220 g	Aβ_1–42_	12	36	Intraperitoneal injection	25; 50; 100	15	Ethology	MWM
											Pathological changes	Neuron (Nissl)
											Oxidative stress	SOD, GSH-Px, and MDA
Wan et al. (2023)	Mice	B6C3-Tg	M	6 months/NA	APP/PS1	10	20	Oral gavage	10; 20	56	Ethology	MWM
											Oxidative stress	SOD, GSH-Px, and MDA
											Apoptosis	Caspase-3 and Bcl-2/Bax
											Neural and synaptic plasticity	BDNF and PSD-95
											Signaling pathways	PERK-eIF-2α, IRE-1α, ATF6, and CHOP
Wen et al. (2014)	Rats	SD	M	NA/260∼300 g	Aβ_1-42_	10	10	Oral gavage	8	30	Apoptosis	Caspase-3
											Signaling pathways	NF-κB and PI3K/Akt
Xiang et al. (2024a)	Rats	SD	M	NA/250∼300 g	STZ	8	24	Oral gavage	20; 40; 80	24	Ethology	MWM
											Pathological changes	Neuron (Nissl)
											Neuroinflammation	TNF-α, IL-1β, and IL-6
											Oxidative stress	SOD and MDA
											Neural and synaptic plasticity	BDNF
											Signaling pathways	CREB-BDNF-TrkB
											Other	NFTs AChE (cholinergic system)
											Oxidative stress	SOD, MDA, and ROS
											Apoptosis	Caspase-3
											Other	AChE and ChAT (cholinergic system)
Yang et al. (2024)	Mice	C57BL/6J	M	7∼8 weeks/NA	Aβ_1-42_	6	12	Intraperitoneal injection	5; 20	28	Pathological changes	Neuron (HE)
											Neuroinflammation	TNF-α, IL-1β, and IL-6
											Oxidative stress	SOD, GSH-Px, MDA, and ROS
											Apoptosis	Bcl-2/Bax
											Signaling pathways	NEAT1/miR-291a-3p/Rab22 and NF-κB

Abbreviations: ACh, acetylcholine; AChE, acetylcholinesterase; Aβ, β-amyloid; Bcl-2/Bax, B-cell lymphoma-2/Bcl-2-associated X protein; BDNF, brain-derived neurotrophic factor; ChAT, choline acetyl transferase; F, female; GSH-Px, glutathione peroxidase; ICR, improved castle road mice; IHC, immunohistochemistry; IL-1β, interleukin-1 beta; IL-6, interleukin-6; KM, Kunming mice; LPS, lipopolysaccharide-induced Alzheimer’s disease model; M, male; MDA, malondialdehyde; MWM, Morris water maze; NA, not applicable; NC, number of the AD control group (AD + vehicle or sham); NE, number of the experimental group (AD + Tan ⅡA); NFTs, neurofibrillary tangles; PSD-95, postsynaptic density-95; ROS, reactive oxygen species; SD, Sprague–Dawley rats; SOD, superoxide dismutase; STZ, streptozotocin-induced Alzheimer’s disease model; Tan ⅡA, tanshinone ⅡA; Th-S, thioflavine S staining; TNF-α, tumor necrosis factor-alpha.

### Quality assessment and risk of bias

**FIGURE 3:**
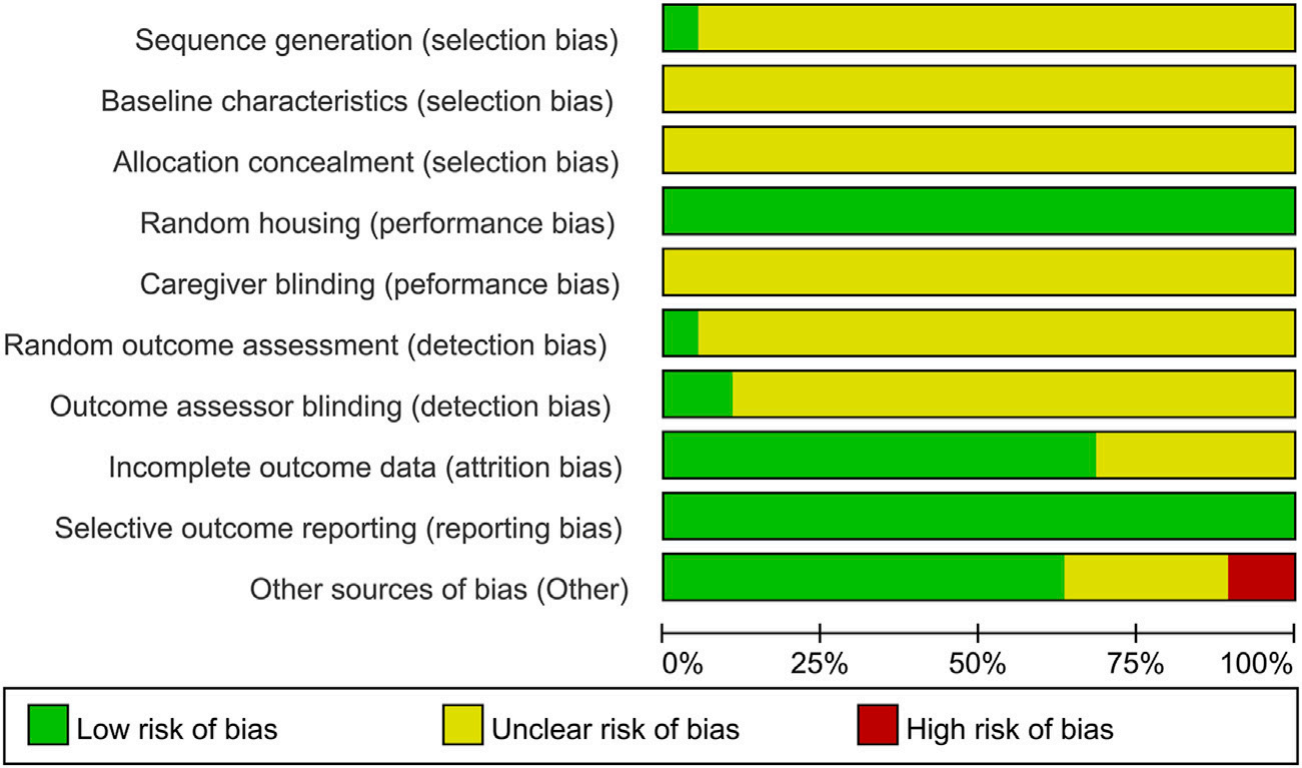
Summary of risk of bias assessment for included studies.

A Cohen’s kappa of 0.72 reflected good agreement between reviewers YR and WS for the risk of bias evaluation using the SYRCLE’s RoB tool. For sequence generation, one study (Lin et al., 2019) used a random number table for randomization, which was considered low risk. The remaining 18 studies claimed randomization without specifying the methods, resulting in an unclear risk rating. As none of the included studies conducted baseline assessments of learning and memory performance following group allocation, the comparability of baseline characteristics across groups could not be determined, leading to an unclear risk rating in this domain. With respect to allocation concealment, the methodological details provided in all studies were insufficient to ascertain whether adequate concealment procedures were implemented, leading to all studies being rated as an unclear risk. Random housing was performed in all studies, which was therefore assessed as low risk. Caregiver blinding was assessed as unclear risk in all included studies. Regarding random outcome assessment, one study (Jiang et al., 2014) was classified as low risk, whereas the other 18 studies were deemed to have unclear risk for the obscure principles governing animal selection. Outcome assessment was processed by observers who were blinded to the experiment design in two studies (Ding et al., 2020; Peng et al., 2022), which had a low risk of bias. The remaining studies were rated as unclear risk. Six studies were rated as unclear risk for incomplete outcome data. Although the study protocols were unavailable, all prespecified outcomes were explicitly reported; thus, all studies were assessed as low risk of reporting bias. For other sources of bias, five studies failed to provide explicit declarations about potential conflicts of interest among co-authors and were assessed as unclear risk. Two studies were assessed as high risk (Wen et al., 2014; Lu et al., 2016) as they exhibited discrepancies between figures and their corresponding legends. In summary, although some studies demonstrated low risk of bias in certain domains, it should be noted that a proportion of studies exhibited unclear or even high risk of bias in specific aspects (Table 2; Figure 3).

**TABLE 2:** Risk of bias using the SYRCLE’s Risk of Bias tool.

Study	Selection bias			Performance bias		Detection bias		Attrition bias (incomplete outcome data)	Reporting bias (selective outcome reporting)	Other (other sources of bias)
	Sequence generation	Baseline characteristic	Allocation concealment	Random housing	Caregiver blinding	Random outcome assessment	Outcome assessor blinding			
Ding et al. (2020)	U	U	U	L	U	U	L	L	L	L
Fang et al. (2021)	U	U	U	L	U	U	U	U	L	U
He et al. (2020a)	U	U	U	L	U	U	U	U	L	U
He et al. (2020b)	U	U	U	L	U	U	U	U	L	L
Jiang et al. (2010)	U	U	U	L	U	U	U	L	L	U
Jiang et al. (2014)	U	U	U	L	U	L	U	L	L	U
Li et al. (2015)	U	U	U	L	U	U	U	L	L	U
Li et al. (2016)	U	U	U	L	U	U	U	L	L	L
Lin et al. (2019)	L	U	U	L	U	U	U	L	L	L
Liu et al. (2016)	U	U	U	L	U	U	U	L	L	L
Liu et al. (2024b)	U	U	U	L	U	U	U	L	L	L
Lu et al. (2016)	U	U	U	L	U	U	U	L	L	H
Luo (2014)	U	U	U	L	U	U	U	L	L	L
Peng et al. (2022)	U	U	U	L	U	U	L	U	L	L
Ren et al. (2024)	U	U	U	L	U	U	U	U	L	L
Wan et al. (2023)	U	U	U	L	U	U	U	U	L	L
Wen et al. (2014)	U	U	U	L	U	U	U	U	L	H
Xiang et al. (2024a)	U	U	U	L	U	U	U	L	L	L
Yang et al. (2024)	U	U	U	L	U	U	U	L	L	L

L, low risk; H, high risk; U, unclear risk.

A Cohen’s kappa of 0.83 reflected an excellent agreement on reporting quality between reviewers YR and WS. Overall, the reporting quality of the included studies was satisfactory as all studies were of high quality with a “Yes” response in more than 70% of parameters. All studies clearly reported timing of intervention, administration route, number of animals per group and defined modeling methods, outcome measures, pharmaceutical preparation, and dosage. However, none of the studies reported model validation as measures of learning and memory were recommended as the ultimate readout (Puzzo et al., 2014). Animal characteristic reporting was incomplete in most studies, typically lacking either age or weight specifications. Furthermore, six studies failed to provide essential information regarding animal dropout (Supplementary Figure S1).

### Effects on cognitive function

A total of 14 studies conducted MWM involving 489 animals, with 340 in the experimental group and 149 in the control group. The escape latency and time spent in the target quadrant were used to evaluate cognitive function.

#### Escape latency of MWM

**FIGURE 4:**
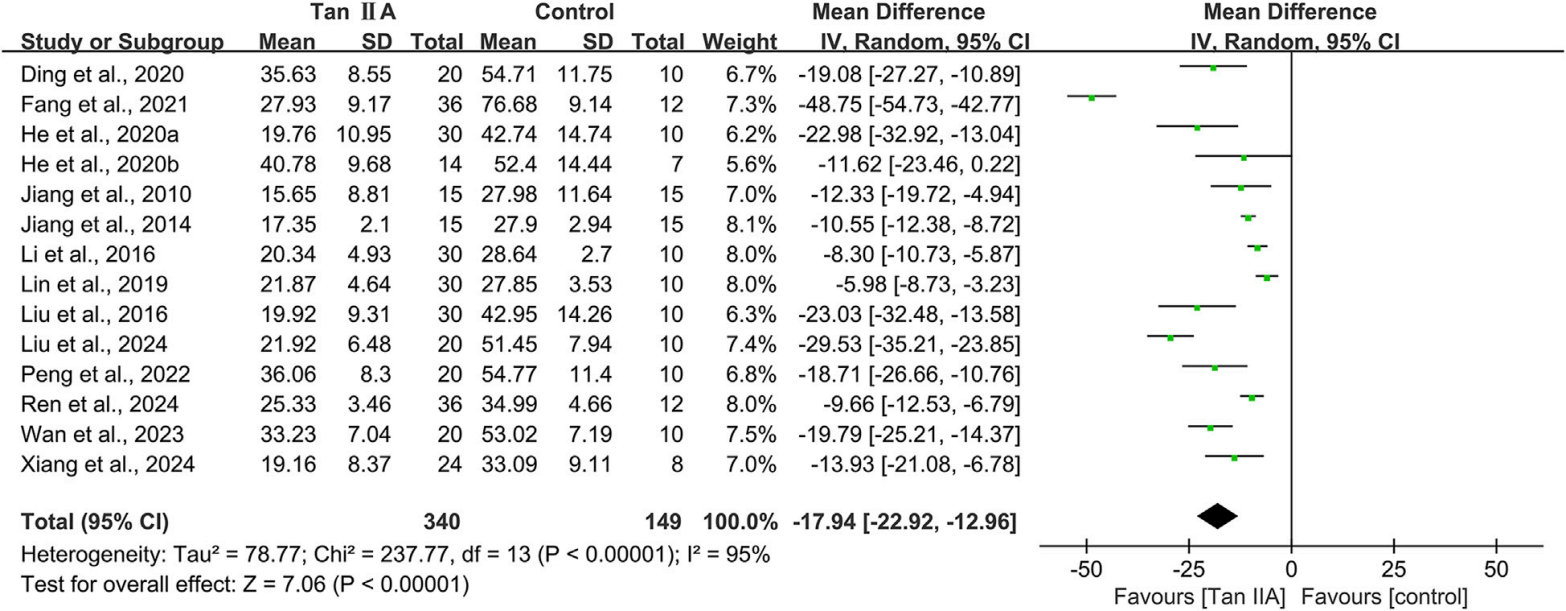
Effect of tanshinone IIA on escape latency of Morris water maze.

The escape latency was reported in all the 14 studies. The pooled result showed a significant reduction in the experimental group with a large effect size (MD = −17.94s; 95% CI: −22.92 to −12.96; *P* < 0.01) and considerable heterogeneity (I^2^ = 95%) compared with that in the control group (Figure 4). Subgroup analysis was conducted according to animal species, model, administration route, dose, and treatment duration (Table 3).

**TABLE 3:** Subgroup analyses of escape latency in Morris water maze.

Subgroup	MD	LL	HL	I^2^	Z	P
Animal species						
Mice	−22.52	−32.21	−12.83	96%	4.55	<0.01
Rats	−9.59	−11.98	−7.19	58%	7.84	<0.01
Model						
Aβ_1–42_	−9.17	−11.66	−6.68	63%	7.22	<0.01
Transgenic AD	−19.52	−27.57	−11.47	92%	4.75	<0.01
STZ	−16.31	−22.78	−9.83	33%	4.94	<0.01
LPS	−48.75	−54.73	−42.77	-	15.99	<0.01
Administration route						
Intraperitoneal injection	−19.88	−29.91	−9.85	96%	3.88	<0.01
Oral gavage	−15.99	−21.62	−10.35	91%	5.56	<0.01
Dose (mg/kg)						
≤40	−26.64	36.81	−16.47	92%	5.13	<0.01
>40	−10.08	−12.33	−7.82	62%	8.77	<0.01
Treatment duration (days)						
≤30	−12.19	−14.80	−9.59	72%	9.19	<0.01
>30	−32.65	−49.13	−16.17	96%	3.88	<0.01

Subgroup analyses based on animal species revealed that although heterogeneity was reduced in the rat subgroup (I^2^ = 58%), it remained substantial in the mice subgroup (I^2^ = 96%). Model-based subgroup analyses indicated minimal heterogeneity in the STZ-induced AD subgroup (I^2^ = 33%), moderate heterogeneity in the Aβ_1–42_-induced AD subgroup (I^2^ = 63%), and considerable heterogeneity in the transgenic AD (APP/PS1) subgroup (I^2^ = 92%). Heterogeneity analysis was not applicable to the LPS-induced AD model due to the inclusion of only one study. Subgroup analyses of the administration route showed high heterogeneity in both the intraperitoneal injection (I^2^ = 96%) and oral gavage (I^2^ = 91%) subgroups. Dose-based subgroup analyses revealed substantial heterogeneity in the ≤40 mg/kg subgroup (I^2^ = 92%) and moderate heterogeneity in the >40 mg/kg subgroup (I^2^ = 62%). Treatment duration-based subgroup analyses indicated moderately high heterogeneity in the ≤30-day subgroup (I^2^ = 72%) and very high heterogeneity in the >30-day subgroup (I^2^ = 96%). These results suggested that the above subgroup classification criteria were not the primary sources of heterogeneity; however, all groups showed statistically significant reductions in escape latency by Tan ⅡA.

#### Time spent in the target quadrant in MWM

**FIGURE 5:**
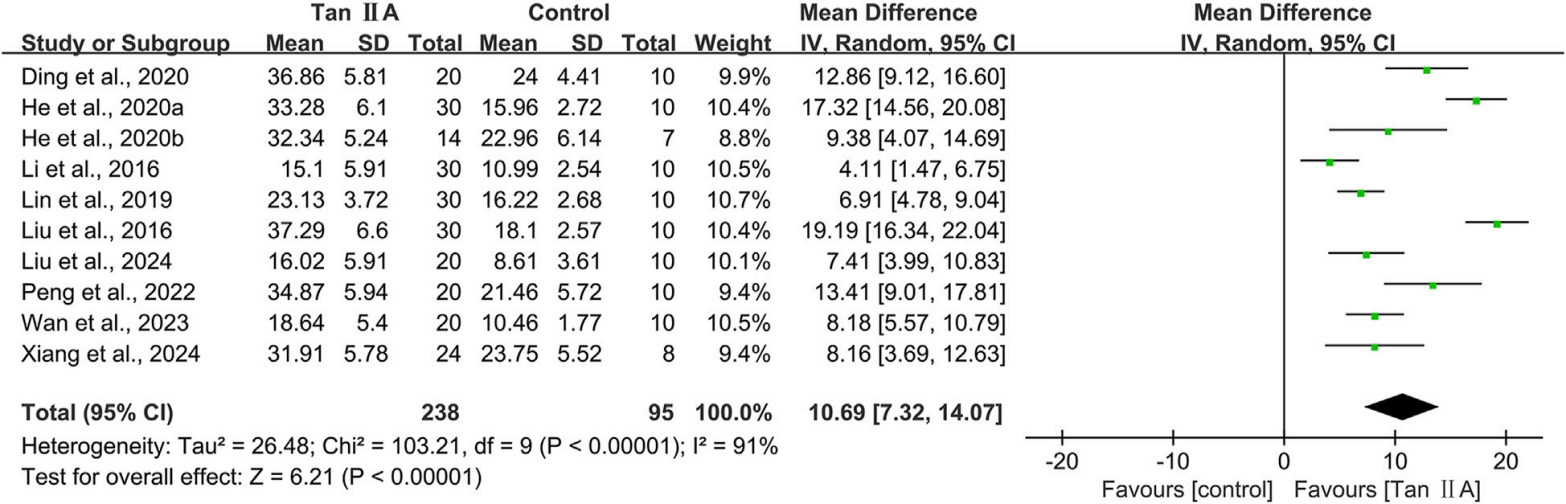
Effect of tanshinone IIA on time spent in the target quadrant in Morris water maze.

The time spent in the target quadrant in MWM was reported in 10 studies. The pooled result showed a significant increase in the Tan ⅡA intervention group with a large effect size (MD = 10.69s; 95% CI: 7.32–14.07; *P* < 0.01) and considerable heterogeneity (I^2^ = 91%) compared with that in the control group (Figure 5). Subgroup analyses were performed based on the same criteria. Similarly, subgroup analyses were performed based on the same classification criteria as described for escape latency (Table 4).

**TABLE 4:** Subgroup analyses of time spent in the target quadrant in Morris water maze.

Subgroup	MD	LL	HL	I^2^	Z	P
Animal species						
Mice	11.50	7.41	15.59	92%	5.51	<0.01
Rats	7.14	5.22	9.06	0%	7.28	<0.01
Model						
Aβ_1–42_	6.91	4.78	9.04	-	6.36	<0.01
Transgenic AD	10.49	6.24	14.73	91%	4.84	<0.01
STZ	12.44	4.60	20.28	91%	3.11	<0.01
Administration route						
Intraperitoneal injection	11.80	5.64	17.97	93%	3.75	<0.01
Oral gavage	9.63	5.55	13.71	90%	4.63	<0.01
Dose (mg/kg)						
≤40	11.81	7.80	15.83	87%	5.77	<0.01
>40	9.55	3.87	15.24	94%	3.29	<0.01
Treatment duration (days)						
≤30	11.44	7.22	15.66	93%	5.31	<0.01
>30	7.90	5.82	9.97	0%	7.46	<0.01

Subgroup analyses by animal species showed that heterogeneity in the mouse subgroup remained virtually unchanged (I^2^ = 92%), whereas the rat subgroup exhibited no heterogeneity (I^2^ = 0%). Model-based subgroup analyses indicated comparable heterogeneity in the transgenic AD (APP/PS1) and STZ-induced AD subgroups (I^2^ = 91% for both). Heterogeneity testing was inapplicable to the Aβ_1–42_-induced AD model due to a single included study. Administration route subgroup analyses revealed substantially high heterogeneity in both the intraperitoneal injection (I^2^ = 93%) and oral gavage (I^2^ = 90%) subgroups. Dose-based subgroup analyses showed considerable heterogeneity in both the ≤40 mg/kg (I^2^ = 87%) and >40 mg/kg (I^2^ = 94%) subgroups. Treatment duration subgroup analyses demonstrated that heterogeneity in the ≤30-day subgroup remained substantially high; however, it decreased significantly in the >30-day subgroup (I^2^ = 0%). These findings suggested that the above subgroup classification criteria were not the primary sources of heterogeneity; however, all groups showed statistically significant prolongation of time spent in the target quadrant by Tan ⅡA.

### Pathological changes

Aβ plaques and subsequent neuronal loss are recognized as two hallmark pathological features of AD. The hippocampal Aβ plaque burden and neuronal damage were used to assess the neuropathological effects of Tan ⅡA on brain tissue.

#### Hippocampal Aβ plaque burden

**FIGURE 6:**
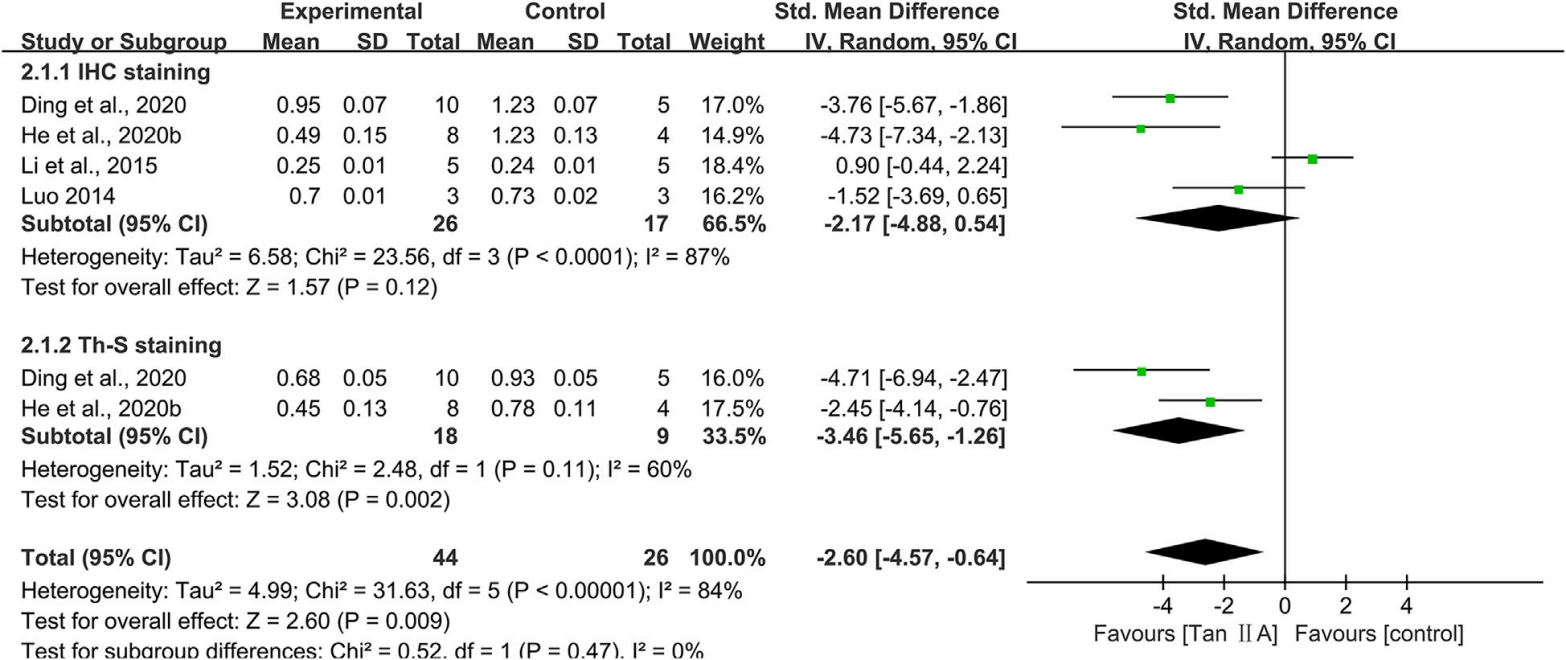
Effect of tanshinone IIA on hippocampal Aβ plaque burden.

Immunohistochemistry across four studies demonstrated a reduction in the percentage area occupied by Aβ plaques, as visualized using specific anti-Aβ antibody staining, following Tan ⅡA treatment (SMD = −2.17; 95% CI: −4.88 to −0.54), with considerable heterogeneity (I^2^ = 87%). However, this reduction failed to reach statistical significance (*P* = 0.12) (Figure 6). One study (Li et al., 2015) reported comparable Aβ plaque levels between the control group and the Tan ⅡA group. After excluding this study, the pooled result of the remaining three studies showed a statistically significant positive effect of Tan ⅡA in reducing Aβ plaque burden (SMD = −3.27; 95% CI: −5.07 to −1.48; *P* < 0.01), with moderate heterogeneity (I^2^ = 50%). This suggested that the pooled results were substantially influenced by this study. Subgroup analyses were conducted according to animal species, model, dose, and treatment duration (Supplementary Table S2), and the results of the meta-analysis were also not robust. Meta-analysis of two studies using thioflavine S staining for Aβ visualization further substantiated the effect of Tan ⅡA (SMD = −3.46; 95% CI: −5.65 to −1.26; *P* < 0.01), with moderate heterogeneity (I^2^ = 60%) (Figure 6).

#### Hippocampal neuronal damage

**FIGURE 7:**
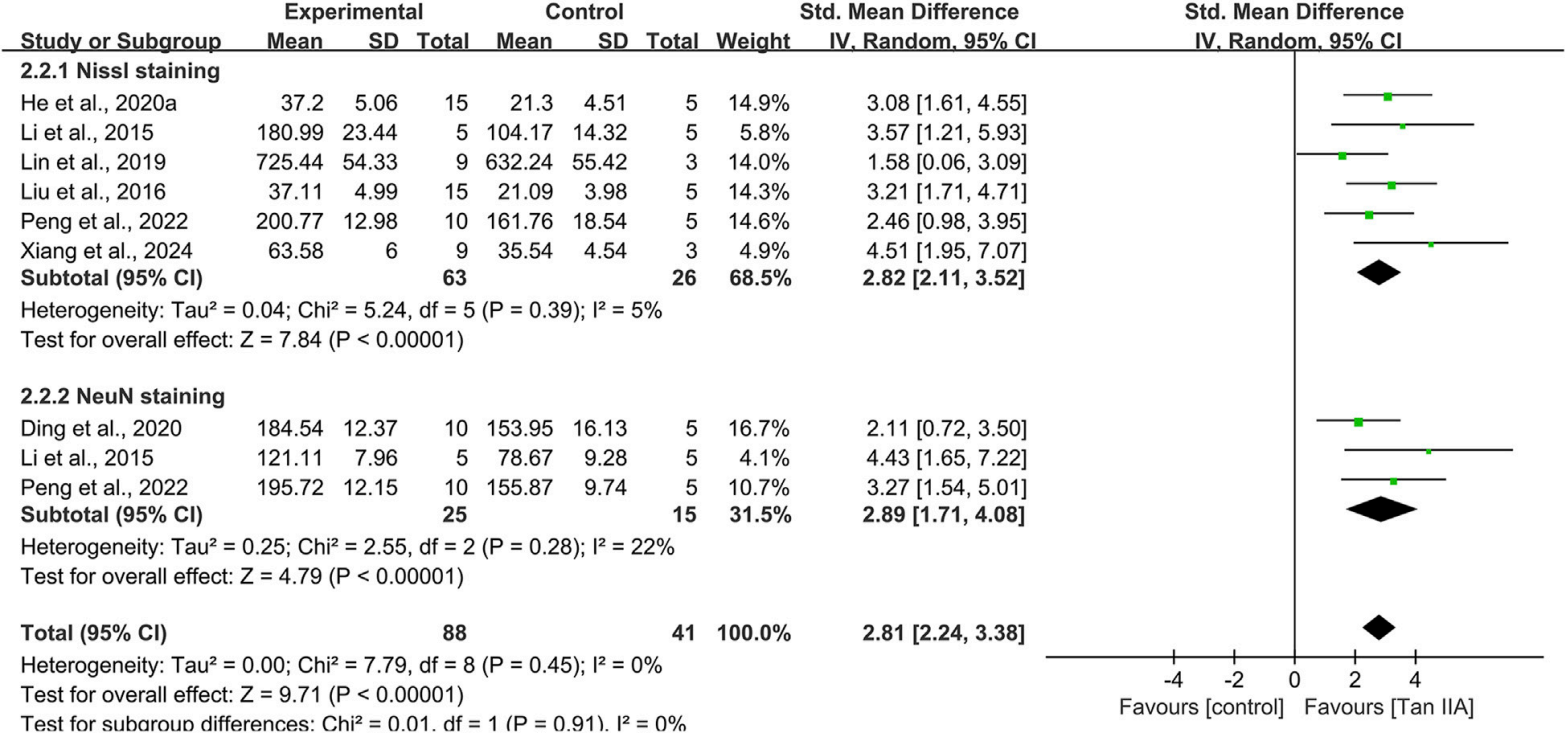
Effect of tanshinone IIA on hippocampal neuronal damage.

Eight studies used Nissl staining to assess hippocampal neuronal damage, with six studies providing quantitative data. The pooled analysis revealed a significant increase in the number of Nissl bodies in experimental groups, with a large effect size (SMD = 2.82; 95% CI: 2.11–3.52; *P* < 0.01) and minimal heterogeneity (I^2^ = 5%) (Figure 7). Two studies (Luo, 2014; Ren et al., 2024) provided narrative evidence of hippocampal neuronal damage in control groups, characterized by reduced Nissl bodies and cytoplasmic atrophy, whereas Tan ⅡA treatment partially reversed these degenerative changes.

Three studies conducted NeuN immunohistochemistry, with pooled results indicating significantly increased NeuN-positive cell counts in experimental groups compared to that in controls, with a large effect size (SMD = 2.89; 95% CI: 1.71–4.08; *P* < 0.01) and acceptable heterogeneity (I^2^ = 22%) (Figure 7).

### Neuroinflammation

Inflammation markers in brain tissue were reported as TNF-α, IL-1β, and IL-6.

#### TNF-α

**FIGURE 8:**
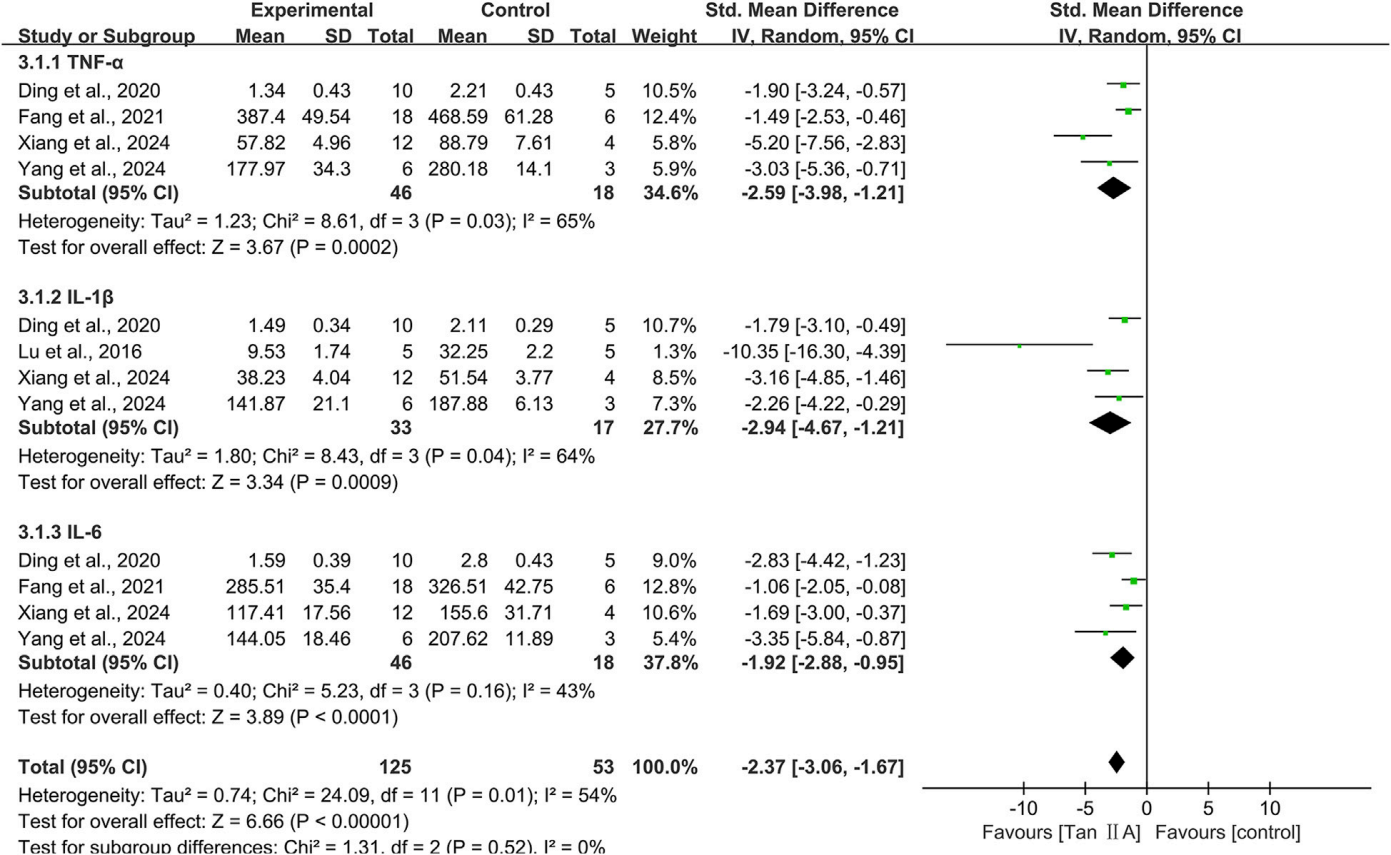
Effect of tanshinone IIA on neuroinflammation.

Four studies investigating the TNF-α level encompassed 64 animals (46 in experimental groups and 18 in controls). The pooled results showed that Tan ⅡA reduced TNF-α levels (SMD = −2.59; 95% CI: −3.98 to −1.21; *P* < 0.01), with moderately high heterogeneity (I^2^ = 65%) (Figure 8). Leave-one-out sensitivity analysis revealed that excluding the study by Xiang et al. (2024b) eliminated heterogeneity (I^2^ = 0%) among the remaining three studies, indicating that this study was likely the source of heterogeneity. Notably, this study differed from the others in administering Tan ⅡA via oral gavage to rat models at an average dose >40 mg/kg. Subgroup analysis was conducted according to animal species, model, administration route, dose, and treatment duration (Supplementary Table S3).

#### IL-1β

Four studies evaluating the IL-1β level included 50 animals (33 in experimental groups and 17 in controls). The pooled results revealed that Tan ⅡA reduced IL-1β levels (SMD = −2.94; 95% CI: −4.67 to −1.21; *P* < 0.01), with moderately high heterogeneity (I^2^ = 64%) (Figure 8). After excluding one study (Lu et al., 2016), the remaining three studies showed homogeneity (I^2^ = 0%), and the overall effect remained statistically significant with minimal change, suggesting that the excluded study was likely the source of heterogeneity. Subgroup analysis was conducted according to animal species, model, administration route, and dose (Supplementary Table S4).

#### IL-6

Four studies identical to those reporting TNF-α levels were included. The pooled results revealed that Tan ⅡA reduced IL-6 levels (SMD = −1.92; 95% CI: −2.88 to −0.95; *P* < 0.01), with moderate-to-low heterogeneity (I^2^ = 43%) (Figure 8). One study (Fang et al., 2021) had a treatment duration of 7 weeks. After excluding this study, heterogeneity decreased significantly (I^2^ = 0%), and the impact on the overall effect was minimal, thereby revealing it as the source of heterogeneity. These four studies were subjected to subgroup analyses using identical stratification criteria as utilized for TNF-α evaluation (Supplementary Table S5).

### Oxidative stress

Oxidative stress biomarkers in brain tissue were evaluated using both antioxidant stress indicators (SOD and GSH-Px) and pro-oxidative stress indicators (MDA and ROS).

#### SOD

The pooled results of seven studies showed statistically significant intergroup differences (SMD = 3.08; 95% CI: 2.21–3.95; *P* < 0.01), with moderate-to-low heterogeneity (I^2^ = 41%) (Supplementary Figure S2). Subgroup analysis was conducted according to animal species, model, administration route, dose, and treatment duration.

Subgroup analyses by animal species revealed moderate heterogeneity in both mouse (I^2^ = 50%) and rat (I^2^ = 54%) subgroups. Model-based subgroup analyses showed that heterogeneity significantly decreased in the Aβ_1–42_-induced AD model subgroup (I^2^ = 14%), whereas it vanished in both transgenic AD (I^2^ = 0%) and STZ-induced AD (I^2^ = 0%) subgroups, suggesting model differences as potential sources of heterogeneity. For the administration route, substantial heterogeneity was observed in the intraperitoneal injection subgroup (I^2^ = 55%), contrasting with the acceptable heterogeneity in the oral gavage subgroup (I^2^ = 36%). Dose-based subgroup analyses indicated homogeneity within the ≤40 mg/kg subgroup (I^2^ = 0%), whereas variations persisted in the >40 mg/kg subgroup (I^2^ = 52%). Subgroup analyses by treatment duration showed minimal heterogeneity in the >30-day subgroup (I^2^ = 14%) and near-unchanged heterogeneity in the ≤30-day subgroup (I^2^ = 42%) (Table 5).

**TABLE 5:** Subgroup analyses of SOD.

Subgroup	SMD	LL	HL	I^2^	Z	P
Animal species						
Mice	3.09	1.94	4.23	50%	5.29	<0.01
Rats	3.17	1.34	5.01	54%	3.39	<0.01
Model						
Aβ_1–42_	3.48	1.89	5.07	14%	4.29	<0.01
Transgenic AD	3.95	2.81	5.09	0%	6.77	<0.01
STZ	2.02	1.09	2.94	0%	4.28	<0.01
Administration route						
Intraperitoneal injection	3.08	1.72	4.44	55%	4.44	<0.01
Oral gavage	3.17	1.89	4.45	36%	4.86	<0.01
Dose (mg/kg)						
≤40	3.66	2.65	4.66	0%	7.11	<0.01
>40	2.59	1.31	3.86	52%	3.98	<0.01
Treatment duration (days)						
≤30	2.85	1.84	3.86	42%	5.54	<0.01
>30	3.77	2.28	5.26	14%	4.97	<0.01

#### GSH-Px

The pooled results of six studies involving 90 animals (63 in experimental groups and 27 in control groups) showed that Tan ⅡA significantly increased GSH-Px (SMD = 2.31; 95% CI: 1.69–2.92; *P* < 0.01), without heterogeneity (I^2^ = 0%) (Supplementary Figure S2).

#### MDA

The pooled results of seven studies showed that Tan ⅡA significantly decreased MDA (SMD = −2.86; 95% CI: −3.67 to −2.05; *P* < 0.01), with acceptable heterogeneity (I^2^ = 35%) (Supplementary Figure S3). Subgroup analysis was conducted according to the animal species, model, administration route, dose, and treatment duration.

Subgroup analyses by animal species showed a significant decrease in heterogeneity in the mouse subgroup (I^2^ = 14%) and an increase in the rat subgroup (I^2^ = 64%). Model-based subgroup analyses revealed that heterogeneity vanished in both Aβ_1–42_-induced AD and STZ-induced AD (I^2^ = 0%) subgroups, whereas it remained near-unchanged in the transgenic AD subgroup (I^2^ = 34%). For the administration route, the oral gavage subgroup showed no heterogeneity (I^2^ = 0%), whereas heterogeneity slightly increased in the intraperitoneal injection subgroup (I^2^ = 39%). Dose-based subgroup analyses indicated a marked increase in heterogeneity in the >40 mg/kg subgroup (I^2^ = 52%), with unchanged heterogeneity in the ≤40 mg/kg subgroup (I^2^ = 35%). Subgroup analyses by treatment duration showed reduced heterogeneity in both ≤30-day (I^2^ = 24%) and >30-day (I^2^ = 0%) subgroups, suggesting that treatment duration might contribute to the observed variations (Table 6).

**TABLE 6:** Subgroup analyses of MDA.

Subgroup	SMD	LL	HL	I^2^	Z	P
Animal species						
Mice	−2.51	−3.30	−1.73	14%	6.27	<0.01
Rats	−3.72	−6.02	−1.42	64%	3.17	<0.01
Model						
Aβ_1–42_	−4.80	−6.63	−2.96	0%	5.12	<0.01
Transgenic AD	−2.51	−3.67	−1.34	34%	4.21	<0.01
STZ	−2.48	−3.48	−1.49	0%	4.88	<0.01
Administration route						
Intraperitoneal injection	−3.50	−4.76	−2.24	39%	5.46	<0.01
Oral gavage	−2.18	−3.01	−1.35	0%	5.15	0.01
Dose (mg/kg)						
≤40	−2.75	−3.91	−1.60	35%	4.66	<0.01
>40	−3.09	−4.48	−1.71	52%	4.38	<0.01
Treatment duration (days)						
≤30	−3.22	−4.16	−2.28	24%	6.72	<0.01
>30	−1.98	−2.95	−1.00	0%	3.96	<0.01

#### ROS

Two studies reported ROS, and the results of meta-analysis showed that Tan ⅡA significantly reduced ROS levels (SMD = −2.23; 95% CI: −4.35 to −0.12; *P* = 0.04), with moderately high heterogeneity (I^2^ = 60%) (Supplementary Figure S3).

### Apoptosis

Western blot results of Caspase-3 and the Bcl-2/Bax ratio were analyzed to evaluate the antiapoptotic effect of Tan ⅡA on AD animal models.

#### Caspase-3

The pooled results of seven studies showed a significant advantage of Tan ⅡA in downregulating Caspase-3 protein expression (SMD = −2.51; 95% CI: −3.22 to −1.79; *P* < 0.01), with acceptable heterogeneity (I^2^ = 31%) (Supplementary Figure S4). Subgroup analysis was conducted according to the animal species, model, administration route, dose, and treatment duration (Supplementary Table S6).

#### Bcl-2/Bax ratio

The combined results of six studies (all in mice) indicated a significant advantage of Tan ⅡA in upregulating the Bcl-2/Bax ratio (SMD = 4.62 95% CI: 2.13–7.12; *P* < 0.01), with considerable heterogeneity (I^2^ = 87%) (Supplementary Figure S4). Subgroup analysis was conducted according to the model, administration route, dose, and treatment duration (Supplementary Table S7); however, neither study population differences nor intervention parameter variations were responsible for heterogeneity. Leave-one-out sensitivity analysis revealed that excluding one study (Liu et al., 2024b) significantly reduced heterogeneity among the remaining five studies; however, it remained non-negligible (I^2^ = 53%), suggesting this study as a potential source of heterogeneity.

### Neural and synaptic plasticity

Neural and synaptic plasticity was assessed through the quantification of PSD-95 and BDNF expressions.

#### PSD-95

Three studies exclusively evaluated PSD-95 expression in mice. The pooled results of three studies showed a significant advantage of Tan ⅡA in upregulating PSD-95 protein expression (SMD = 5.39; 95% CI: 1.62–9.17; *P* < 0.01), with considerable heterogeneity (I^2^ = 84%) (Supplementary Figure S5). There was evidence suggesting that the study by Liu et al. (2024b) was likely the source of heterogeneity, given that its exclusion led to homogeneous results (I^2^ = 0%) with a stable overall effect.

#### BDNF

The combined results of five studies indicated a significant advantage of Tan ⅡA in enhancing BDNF expression in brain tissue compared to controls (SMD = 2.85; 95% CI: 1.11–4.58; *P* < 0.01), with considerable heterogeneity (I^2^ = 83%) (Supplementary Figure S5). Subgroup analysis was conducted according to the animal species, model, administration route, dose, and treatment duration. Subgroup analyses based on the animal species, model, administration route, dose, and treatment duration indicated that variations in these populations and interventions did not contribute to heterogeneity (Supplementary Table S8). However, heterogeneity vanished (I^2^ = 0%) following the exclusion of the study by Liu et al. (2024a), whereas the overall effect remained essentially unchanged, suggesting the role of this study in driving heterogeneity.

### Meta-regression

Meta-regression was performed for escape latency, time spent in the target quadrant, SOD, and MDA to examine the impact of study quality (risk of bias assessment scores), population characteristics (animal species and model type), and intervention features (administration route, dose, and treatment duration) as covariates on the overall effect, thereby exploring heterogeneity. The meta-regression results for escape latency showed that only treatment duration significantly influenced the effect size (*P* < 0.05), indicating it as a potential factor contributing to heterogeneity. This finding was inconsistent with the results of the subgroup analysis, possibly due to residual confounding factors that prevented complete elimination of heterogeneity within subgroups (Supplementary Table S9). Meta-regression of time spent in the target quadrant, SOD, and MDA failed to detect any significant association, indicating that the factors examined may not account for the heterogeneity among studies (Supplementary Tables S10–S12).

### Publication bias

**FIGURE 9:**
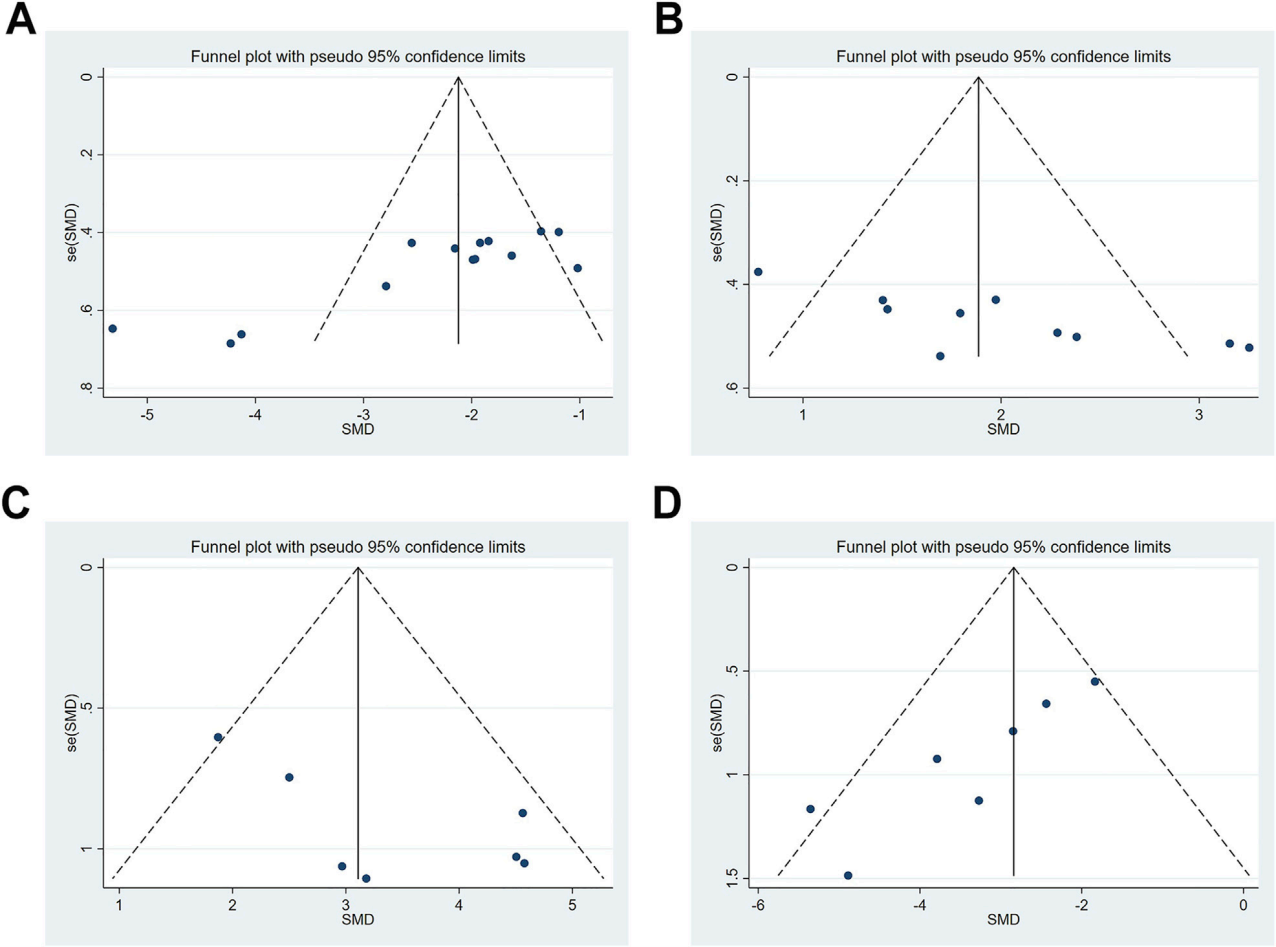
Publication bias represented by funnel plots. **(A)** escape latency; **(B)** time spent in the target quadrant; **(C)** SOD; **(D)** MDA.

Publication bias was evaluated for escape latency, time spent in the target quadrant, SOD, and MDA. Except SOD, visual inspection of the funnel plots revealed evident asymmetry in the distribution of data points. Notably, some points fell outside the 95% confidence intervals, further suggesting the presence of potential publication bias (Figure 9). This observation was statistically confirmed through Egger’s test for escape latency, target quadrant occupancy, and MDA (all *P* < 0.05); however, SOD showed borderline significance (*P* = 0.054). The trim-and-fill method was used to correct funnel plot asymmetry caused by publication bias, followed by repeated meta-analysis using fixed- and random-effects models. Notably, no statistically significant differences were observed in pooled effect sizes before and after trimming, which indicated minimal impact of publication bias and robust results (Supplementary Figure S6; Table 7).

**TABLE 7:** Results from Egger’s test and trim and fill analysis.

Outcome	Egger’s test	Before trim and fill			After trim and fill		
	*P*-value	*P*-value	Est (F/R)	No. of studies	P-value	Est (F/R)	No. of studies
Escape latency	0.000	0.000	−2.122/-2.343	14	0.000	0.120/0.096	14
Time spent in the target quadrant	0.000	0.000	1.886/1.973	10	0.000	4.722/4.872	13
SOD	0.000	0.000	3.109/3.308	7	0.000	9.574/9.986	11
MDA	0.000	0.000	−2.841/-3.136	7	0.000	0.058/0.043	7

### Sensitivity analysis

**FIGURE 10:**
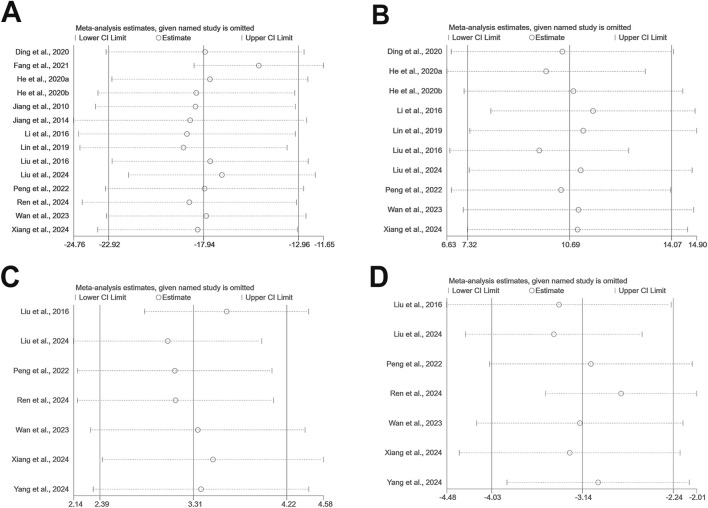
The sensitivity analysis of included studies; **(A)** escape latency; **(B)** time spent in the target quadrant; **(C)** SOD; **(D)** MDA.

Sensitivity analyses were performed using Stata 15.0 software for outcomes including escape latency, time spent in the target quadrant, SOD, and MDA levels to explore the impact of individual studies on the overall effect (Figure 10). The results revealed that the exclusion of any single study had negligible effects on the pooled effect sizes, supporting the consistency and reliability of the meta-analysis outcomes.

## Discussion

### Summary of evidence

To our knowledge, no previous SR/MA has comprehensively evaluated the preclinical efficacy of Tan ⅡA in AD models. Herein, we conducted this work. A total of 19 *in vivo* studies involving 581 AD animals were included, all of which were conducted in China between 2010 and 2024, with the majority published in English. The overall reporting quality was comprehensive and standardized, whereas methodological assessment revealed potential risks of bias. Results of the present study showed that Tan ⅡA significantly improved cognitive performance, as indicated by reduced escape latency and increased target quadrant retention time in the MWM test. Furthermore, Tan ⅡA treatment attenuated AD-related neuropathological changes, evidenced by reduced hippocampal Aβ plaque burden and neuronal damage in brain tissue. At the molecular level, Tan ⅡA significantly downregulated pro-inflammatory cytokines (TNF-α, IL-1β, and IL-6). The compounds also exhibited potent antioxidant properties, as demonstrated by decreased MDA and ROS levels and increased SOD and GSH-Px levels. Meanwhile, Tan ⅡA displayed notable antiapoptotic effects through Caspase-3 suppression and Bcl-2/Bax ratio elevation. Additionally, treatment upregulated neural and synaptic plasticity markers (PSD-95 and BDNF), suggesting the potential of Tan ⅡA for neural and synaptic restoration in AD pathology.

### Possible mechanisms of Tan ⅡA in AD

**FIGURE 11:**
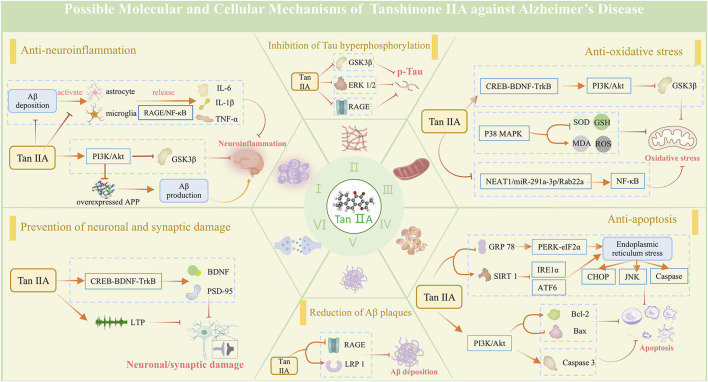
Possible mechanisms of tanshinone IIA against Alzheimer's disease. The neuroprotective mechanisms of tanshinone IIA are associated with its effects in anti-neuroinflammation, anti-oxidative stress, anti-apoptosis, and prevention of neuronal and synaptic damage, along with its ability to reduce Aβ plaques and inhibit Tau protein hyperphosphorylation.

The observed outcomes in this study corroborate earlier findings that Tan ⅡA exhibits neuroprotective effects in AD treatment (Subedi and Gaire, 2021; Sherawat and Mehan, 2023), primarily through modulation of inflammation, oxidative stress, apoptosis, synaptic plasticity, Aβ aggregation, and Tau hyperphosphorylation (Figure 11).

Elevated inflammatory cytokines have been observed in the brain of patients with early-stage AD, suggesting a significant contribution of neuroinflammation to AD pathology (Heneka et al., 2015). Aβ peptide accumulation induces chronic microglia activation, triggering the inflammatory cascade by eliciting expressions of pro-inflammatory cytokines, including TNF-α, IL-1β, and IL-6. These cytokines subsequently contribute to neuronal dysfunction, apoptosis, and synaptic loss (Leng and Edison, 2021; Thakur et al., 2023). Tan ⅡA can suppress glial activation and subsequent neuroinflammatory responses by downregulating Iba-1 and GFAP expressions, an effect partially attributed to its inhibition of the RAGE/NF-κB signaling cascade (Ding et al., 2020). Additionally, Tan ⅡA exerts anti-inflammatory effects via upregulation of PI3K/AKT phosphorylation (Fang et al., 2021).

Compared with other organs, the brain is particularly susceptible to oxidative stress damage. The imbalance between oxidation and antioxidation generates an excess of ROS, leading to damage to lipids, proteins, and DNA, which may underlie the cognitive impairment in AD (Butterfield et al., 2001). MDA, which is the end product of lipid peroxidation, exerts severe cytotoxic effects. SOD, the first line of defense against oxidative damage, and GSH-Px, a key antioxidant enzyme in the body, work together to protect the brain from oxidative stress. Tan ⅡA can suppress oxidative stress-induced brain tissue damage and promote cognitive function recovery. Mechanistically, these effects are associated with the activation of the CREB-BDNF-TrkB pathway (Xiang et al., 2024a), stimulation of the PI3K/Akt/GSK3β pathway (Peng et al., 2022; Liu et al., 2024b), and inhibition of the p38 MAPK pathway (Liu et al., 2016), thereby enhancing the activity of endogenous antioxidants (SOD and GSH), reducing MDA levels, and scavenging ROS.

Neuronal apoptosis compromises brain structure and function. Postmortem studies have identified distinct apoptotic features in AD brains, including altered expression profiles of proapoptotic and antiapoptotic regulators in brain extracts (Kitamura et al., 1998). Endoplasmic reticulum stress (ERS) represents a critical apoptotic pathway, triggering the unfolded protein response (UPR) which either orchestrates adaptive programs to restore homeostasis or triggers apoptosis of irreversibly damaged cells (Gerakis and Hetz, 2018). Tan ⅡA exerts antiapoptotic effects through multiple mechanisms: a. by downregulating GRP78, a core ERS chaperone protein, and suppressing PERK/eIF2α, IRE1α, ATF6, CHOP, and JNK signaling pathways to mitigate ERS-induced apoptosis (He et al., 2020a; He et al., 2020b); b. through PI3K/Akt-mediated regulation of apoptotic proteins, suppressing Bax while enhancing Bcl-2 expression (Liu et al., 2024b); and c. by inhibiting caspase cascade activation, particularly preventing Caspase-3 activation, thereby attenuating apoptotic progression (Wen et al., 2014; He et al., 2020a; He et al., 2020b).

Neuronal death or a reduction in neuronal density is observed in brain regions critically associated with memory function in AD patients (Miller et al., 2022). Neuronal loss in AD initiates during the preclinical stage and progressively advances through the prodromal phase, including mild cognitive impairment (MCI), ultimately culminating in dementia. Synaptic degeneration, serving as an early harbinger of neuronal degeneration, was discovered to typically precede neuronal loss, with both pathological mechanisms synergistically contributing to AD-associated cognitive dysfunction (Abdi et al., 2022; Koch and Spampinato, 2022). Neuronal and synaptic functions are regulated by a complex interplay of neurotransmitters, neurotrophic factors, and associated proteins. Tan ⅡA can promote synaptogenesis and enhance neuronal plasticity by upregulating synaptic proteins SYN and PSD-95 and activating BDNF (Ding et al., 2020; Liu et al., 2024b). Moreover, it demonstrates neuroprotective properties by rescuing long-term potentiation defects, which is associated with the activation of the CREB-BDNF-TrkB pathway (Xiang et al., 2024a).

Aβ plaque and NFT accumulation, the two typical pathological hallmarks of AD, synergize with neuroinflammation, oxidative stress, apoptosis, and synaptic damage, ultimately resulting in cognitive decline. Tan IIA not only indirectly reduces Aβ deposition and NFT formation by ameliorating the above processes but also exerts direct effects. It enhances Aβ degradation by upregulating IDE and NEP (Liu et al., 2024b), two pivotal Aβ-degrading enzymes with dual intracellular and extracellular activities. It has been shown that Tan IIA can upregulate sAPPα expression, which is a cleavage product of APP by α-secretase, implying a reduction in Aβ production via the β-secretase pathway (Zhang et al., 2020). Additionally, it modulates the RAGE/LRP1 transport system on vascular endothelial cells to accelerate trans-endothelial clearance of Aβ (Wan et al., 2023). In the formation and aggregation of NFTs, Tan IIA suppresses Tau hyperphosphorylation by inhibiting the activation of GSK-3β and ERK (Lin et al., 2019). An *in vitro* experiment demonstrates that Tan IIA induces Tau polyubiquitination, leading to its proteasomal degradation. Meanwhile, Tan IIA can bind to Tau protein to inhibit the formation of Tau fibrils (Cai et al., 2020).

### Limitations

First, the preponderance of included studies conducted in China potentially introduces geographical bias into these meta-analysis results. We advocate for multicenter, international studies from different geographical regions. Moreover, studies should standardize experimental protocols to enhance repeatability and comparability of results. By incorporating a broader range of contexts, future research can generate more globally applicable evidence for Tan IIA’s efficacy in AD treatment.

Second, the evaluation of risk of bias and methodological quality uncovered significant flaws in research implementation, especially unclear risks regarding blinded outcome assessors and the lack of model validation in most studies. Failure to apply the blinding method is likely to lead to an overestimation of the effect size of Tan IIA. Model validation based on pathology or ethology is the foundation for simulating the clinical features of AD and evaluating the effect of Tan IIA. These limitations threaten the stability of results and the generalizability of the conclusion, warranting cautious interpretation. In future experimental studies, scientific and rigorous protocols should be formulated with reference to the guidelines, and the principles of randomization and blinding should be followed to reduce bias and strengthen experimental validity.

Finally, the limitations of this SR/MA must be acknowledged. PICO-related heterogeneity across included studies, such as varied animal models, intervention protocols, and outcome assessments, may limit the reliability of synthesized results. Despite efforts to retrieve grey literature, no additional studies were available. The absence of unpublished research, including negative, ongoing, and industry-sponsored studies, compromised the comprehensiveness of the search. Future studies should expand the search scope, implement rigorous literature screening and bias assessment, and adopt advanced statistical methods to address heterogeneity, thereby enhancing the quality and credibility of the findings.

### Safety and toxicity of Tan ⅡA

Tan IIA may exert side effects in humans or animals. Safety outcomes were derived from 22 clinical studies using sodium tanshinone IIA sulfonate (STS) injection (Wang et al., 2014). Among 27 cases, skin and adnexa injuries (e.g., rash) accounted for 29.9%, systemic damages (e.g., anaphylactic shock) accounted for 21.5%, circulatory system injuries (e.g., chest tightness) accounted for 20.6%, and central/peripheral nervous system injuries (e.g., dizziness) accounted for 15.9%. All the same, these adverse reactions resolved or subsided after symptomatic treatment, without obvious sequelae. We noted that a zebrafish model study demonstrated developmental, cardiovascular, and neurotoxicity of Tan ⅡA at 40 μM concentration (Lai, 2020). In contrast, STS injection at clinical concentrations (1 mg/ml) showed no hemolytic or erythrocyte agglutination effects in guinea pigs, and equivalent doses (4 mg/kg) exhibited no irritant effects on rabbit auricular veins or quadriceps muscles, nor did it induce systemic allergic reactions in guinea pigs (Cao et al., 2010). Notably, none of the included studies in this SR/MA measured safety for Tan IIA in AD treatment, posing challenges to clinical translation of our findings. Given Tan IIA’s potential for AD, future research should focus on safety evaluation to determine safe dosage, concentration, and administration frequency.

## Conclusion

This SR/MA synthesizes preclinical evidence regarding the therapeutic potential of Tan ⅡA in AD rodent models. The analysis suggests that Tan ⅡA demonstrates neuroprotective properties, ameliorating cognitive deficits and attenuating neuropathological alterations in AD progression. These effects are likely mediated through multiple mechanisms, including anti-neuroinflammatory actions, antioxidant stress effects, antiapoptotic properties, synaptic plasticity enhancement, and reversal of neuronal damage. Nevertheless, the presence of heterogeneity and potential bias warrants careful consideration, as they may impact the robustness and reliability of the study outcomes. Future research should focus on conducting rigorously designed animal studies to further investigate the efficacy and safety of Tan ⅡA for AD, followed by clinical validation through well-controlled trials for translating these findings into clinical practice.

## Data Availability

The original contributions presented in the study are included in the article/Supplementary Material; further inquiries can be directed to the corresponding authors.

## Abbreviations

AD, Alzheimer’s disease; Aβ, β-amyloid; Bcl-2/Bax, B-cell lymphoma-2/Bcl-2-associated X protein; BDNF, brain-derived neurotrophic factor; ERS, endoplasmic reticulum stress; GSH-Px, glutathione peroxidase; ICR, improved castle road mice; IL-1β, interleukin-1 beta; IL-6, interleukin-6; LPS, lipopolysaccharide-induced AD model; MA, meta-analysis; MD, mean difference; MDA, malondialdehyde; MWM, Morris water maze; NFTs, neurofibrillary tangles; PSD-95, postsynaptic density-95; ROS, reactive oxygen species; SD, standard deviation; SD rats, Sprague–Dawley rats; SEM, standard error of the mean; SMD, standardized mean difference; SOD, superoxide dismutase; SR, systematic review; STS, sodium tanshinone IIA sulfonate; STZ, streptozotocin-induced AD model; Tan ⅡA, tanshinone ⅡA; TCM, traditional Chinese medicine; TNF-α, tumor necrosis factor-alpha; UPR, unfolded protein response.

## References

[B1] Abdi S. Javanmehr N. Ghasemi-Kasman M. Bali H. Y. Pirzadeh M. (2022). Stem cell-based therapeutic and diagnostic approaches in Alzheimer's disease. Curr. Neuropharmacol. 20 (6), 1093–1115. 10.2174/1570159x20666211231090659 34970956 PMC9886816

[B2] Arefnezhad R. Nejabat A. Behjati F. Torkamanche M. Zarei H. Yekkehbash M. (2024). Tanshinone IIA against cerebral ischemic stroke and ischemia-reperfusion injury: a review of the current documents. Mini Rev. Med. Chem. 24, 1701–1709. 10.2174/0113895575299721240227070032 38482618

[B3] Butterfield D. A. Drake J. Pocernich C. Castegna A. (2001). Evidence of oxidative damage in Alzheimer's disease brain: central role for amyloid beta-peptide. Trends Mol. Med. 7 (12), 548–554. 10.1016/s1471-4914(01)02173-6 11733217

[B4] Cai N. Chen J. Bi D. Gu L. Yao L. Li X. (2020). Specific degradation of endogenous Tau protein and inhibition of Tau fibrillation by tanshinone IIA through the ubiquitin-proteasome pathway. J. Agric. Food Chem. 68 (7), 2054–2062. 10.1021/acs.jafc.9b07022 31995984

[B5] Cao X. M. Chen X. M. Sun W. L. (2010). Safety of sodium tanshinone ⅡA sulfonate injection. J. Med. Res. & Combat Trauma Care 23 (05), 474–476. 10.16571/j.cnki.1008-8199.2010.05.003

[B6] Cecerska-Heryć E. Polikowska A. Serwin N. Roszak M. Grygorcewicz B. Heryć R. (2022). Importance of oxidative stress in the pathogenesis, diagnosis, and monitoring of patients with neuropsychiatric disorders, a review. Neurochem. Int. 153, 105269. 10.1016/j.neuint.2021.105269 34971747

[B7] Cummings J. Zhou Y. Lee G. Zhong K. Fonseca J. Cheng F. (2024). Alzheimer's disease drug development pipeline: 2024. Alzheimers Dement. (N Y) 10 (2), e12465. 10.1002/trc2.12465 38659717 PMC11040692

[B8] Ding B. Lin C. Liu Q. He Y. Ruganzu J. B. Jin H. (2020). Tanshinone IIA attenuates neuroinflammation via inhibiting RAGE/NF-κB signaling pathway *in vivo* and *in vitro* . J. Neuroinflammation 17 (1), 302. 10.1186/s12974-020-01981-4 33054814 PMC7559789

[B9] Driscoll I. F. Lose S. Ma Y. Bendlin B. B. Gallagher C. Johnson S. C. (2024). KLOTHO KL-VS heterozygosity is associated with diminished age-related neuroinflammation, neurodegeneration, and synaptic dysfunction in older cognitively unimpaired adults. Alzheimers Dement. 20, 5347–5356. 10.1002/alz.13912 39030746 PMC11350058

[B10] Fang L. Cheng H. Chen W. Peng C. Liu Y. Zhang C. (2024). Therapeutic effects of Tanshinone IIA and Tetramethylpyrazine nanoemulsions on cognitive impairment and neuronal damage in Alzheimer's disease rat models. J. Pharm. Pharmacol. 76, 1169–1177. 10.1093/jpp/rgae069 38934298

[B11] Fang Y. Song G. J. Chen L. Feng J. Q. (2021). Neuroprotective effect of tanshinone ⅡA and its effects on the PI3K/AKT pathway in a mouse model of Alzheimer's disease. Zhongguo Shiyan Dongwu Xuebao 29 (04), 499–505. 10.3969/j.issn.1005-4847.2021.04.011

[B12] Gerakis Y. Hetz C. (2018). Emerging roles of ER stress in the etiology and pathogenesis of Alzheimer's disease. Febs J. 285 (6), 995–1011. 10.1111/febs.14332 29148236

[B13] Gustavsson A. Norton N. Fast T. Frölich L. Georges J. Holzapfel D. (2023). Global estimates on the number of persons across the Alzheimer's disease continuum. Alzheimers Dement. 19 (2), 658–670. 10.1002/alz.12694 35652476

[B14] He Y. Jin H. Ji S. Qian Y. Zheng Q. Wu X. (2020a). Tanshinone ⅡA improves memory deficits by inhibiting the endoplasmic reticulum stress and apoptosis in Alzheimer's disease mice. J. Xi'an Jiaot. Univ. Med. Sci. 41 (2), 287–293. 10.7652/jdyxb202002025

[B15] He Y. Ruganzu J. B. Lin C. Ding B. Zheng Q. Wu X. (2020b). Tanshinone IIA ameliorates cognitive deficits by inhibiting endoplasmic reticulum stress-induced apoptosis in APP/PS1 transgenic mice. Neurochem. Int. 133, 104610. 10.1016/j.neuint.2019.104610 31778727

[B16] Heneka M. T. Carson M. J. El Khoury J. Landreth G. E. Brosseron F. Feinstein D. L. (2015). Neuroinflammation in Alzheimer's disease. Lancet Neurol. 14 (4), 388–405. 10.1016/s1474-4422(15)70016-5 25792098 PMC5909703

[B17] Hooijmans C. R. Rovers M. M. de Vries R. B. Leenaars M. Ritskes-Hoitinga M. Langendam M. W. (2014). SYRCLE's risk of bias tool for animal studies. BMC Med. Res. Methodol. 14, 43. 10.1186/1471-2288-14-43 24667063 PMC4230647

[B18] Huang L. K. Kuan Y. C. Lin H. W. Hu C. J. (2023). Clinical trials of new drugs for Alzheimer disease: a 2020-2023 update. J. Biomed. Sci. 30 (1), 83. 10.1186/s12929-023-00976-6 37784171 PMC10544555

[B19] Jiang P. Chen M. Lv J. Chen C. Jiao B. H. (2010). Effect of tanshinone Ⅱ A on MMP-2 and iNOS expression and free radical release in hippocampus of rat Alzheimer's disease model. Haijun Junyi Daxue Xuebao 31 (04), 380–384. 10.3724/SP.J.1008.2010.00380

[B20] Jiang P. Li C. Xiang Z. Jiao B. (2014). Tanshinone IIA reduces the risk of Alzheimer's disease by inhibiting iNOS, MMP‑2 and NF‑κBp65 transcription and translation in the temporal lobes of rat models of Alzheimer's disease. Mol. Med. Rep. 10 (2), 689–694. 10.3892/mmr.2014.2254 24859152

[B21] Kitamura Y. Shimohama S. Kamoshima W. Ota T. Matsuoka Y. Nomura Y. (1998). Alteration of proteins regulating apoptosis, bcl-2, bcl-x, Bax, bak, bad, ICH-1 and CPP32, in Alzheimer's disease. Brain Res. 780 (2), 260–269. 10.1016/s0006-8993(97)01202-x 9507158

[B22] Koch G. Spampinato D. (2022). Alzheimer disease and neuroplasticity. Handb. Clin. Neurol. 184, 473–479. 10.1016/b978-0-12-819410-2.00027-8 35034755

[B23] Lai S. F. (2020). Bsed on zebrafish model, safety evaluation of tanshinone ⅡA and its derivatives and exploration of the efficacy of phenanthrazole derivatives. China: Guangdong Pharmaceutical University. dissertation/master’s thesis.

[B24] Leng F. Edison P. (2021). Neuroinflammation and microglial activation in Alzheimer disease: where do we go from here? Nat. Rev. Neurol. 17 (3), 157–172. 10.1038/s41582-020-00435-y 33318676

[B25] Li F. Han G. Wu K. (2016). Tanshinone IIA alleviates the AD phenotypes in APP and PS1 transgenic mice. BioMed Res. Int. 2016, 7631801. 10.1155/2016/7631801 27274990 PMC4870344

[B26] Li J. Wang F. Zhou J. Li W. (2015). Effects of tanshinone ⅡA on the expressions of p53, pp53 and apoptosis in the rats with Alzheimer's disease. J. Central South Univ. Med. Sci. 40 (11), 1210–1216. 10.11817/j.issn.1672-7347.2015.11.008 26643424

[B27] Lin L. Jadoon S. S. Liu S. Z. Zhang R. Y. Li F. Zhang M. Y. (2019). Tanshinone IIA ameliorates spatial learning and memory deficits by inhibiting the activity of ERK and GSK-3β. J. Geriatric Psychiatry Neurology 32 (3), 152–163. 10.1177/0891988719837373 30885037

[B28] Liu C. Wu Y. Zha S. Liu M. Wang Y. Yang G. (2016). Treatment effects of tanshinone IIA against intracerebroventricular streptozotocin induced memory deficits in mice. Brain Res. 1631, 137–146. 10.1016/j.brainres.2015.11.040 26656068

[B29] Liu M. Di Y. M. May B. Zhang A. L. Zhang L. Chen J. (2024a). Renal protective effects and mechanisms of Astragalus membranaceus for diabetic kidney disease in animal models: an updated systematic review and meta-analysis. Phytomedicine 129, 155646. 10.1016/j.phymed.2024.155646 38733903

[B30] Liu X. Q. Hu T. Wu G. L. Qiao L. J. Cai Y. F. Wang Q. (2024b). Tanshinone IIA, the key compound in Salvia miltiorrhiza, improves cognitive impairment by upregulating Aβ-degrading enzymes in APP/PS1 mice. Int. J. Biol. Macromol. 254, 127923. 10.1016/j.ijbiomac.2023.127923 37944734

[B31] Lu B. L. Li J. Zhou J. Li W. W. Wu H. F. (2016). Tanshinone IIA decreases the levels of inflammation induced by Aβ1-42 in brain tissues of Alzheimer's disease model rats. NeuroReport 27 (12), 883–893. 10.1097/WNR.0000000000000618 27348015

[B32] Luo H. (2014). TanⅡA effects on the expression of complement C1q and C3 in APP/PS1 transgenic mice. China: Central South University. dissertation/master’s thesis.

[B33] Ma M. Hua X. Jia C. Xiao N. Zhang L. Wei L. (2024). Tanshinone IIA regulates synaptic plasticity in Mg(2+)-free-induced epileptic hippocampal neurons via the PI3K/akt signaling pathway. J. Integr. Neurosci. 23 (3), 61. 10.31083/j.jin2303061 38538223

[B34] Miller M. B. Huang A. Y. Kim J. Zhou Z. Kirkham S. L. Maury E. A. (2022). Somatic genomic changes in single Alzheimer's disease neurons. Nature 604 (7907), 714–722. 10.1038/s41586-022-04640-1 35444284 PMC9357465

[B35] Moher D. Liberati A. Tetzlaff J. Altman D. G. PRISMA Group (2010). Preferred reporting items for systematic reviews and meta-analyses: the PRISMA statement. Int. J. Surg. 8 (5), 336–341. 10.1016/j.ijsu.2010.02.007 20171303

[B36] Moreira N. Lima J. Marchiori M. F. Carvalho I. Sakamoto-Hojo E. T. (2022). Neuroprotective effects of cholinesterase inhibitors: current scenario in therapies for Alzheimer's disease and future perspectives. J. Alzheimers Dis. Rep. 6 (1), 177–193. 10.3233/adr-210061 35591949 PMC9108627

[B37] Peng X. Chen L. Wang Z. He Y. Ruganzu J. B. Guo H. (2022). Tanshinone IIA regulates glycogen synthase kinase-3β-related signaling pathway and ameliorates memory impairment in APP/PS1 transgenic mice. Eur. J. Pharmacol. 918, 174772. 10.1016/j.ejphar.2022.174772 35090935

[B38] Puzzo D. Lee L. Palmeri A. Calabrese G. Arancio O. (2014). Behavioral assays with mouse models of Alzheimer's disease: practical considerations and guidelines. Biochem. Pharmacol. 88 (4), 450–467. 10.1016/j.bcp.2014.01.011 24462904 PMC4014001

[B39] Ren W. J. Li X. X. Wang T. Q. Liu Y. X. Dai X. L. Huo Q. (2024). Effects of tanshinone IIA on cognitive impairment in Alzheimer’s disease rats via oxidative stress, inflammatory responses and apoptosis. Pharmacogn. Mag. 20, 1226–1236. 10.1177/09731296241246632

[B40] Saha S. Buttari B. Profumo E. Tucci P. Saso L. (2021). A perspective on Nrf2 signaling pathway for neuroinflammation: a potential therapeutic target in Alzheimer's and Parkinson's diseases. Front. Cell Neurosci. 15, 787258. 10.3389/fncel.2021.787258 35126058 PMC8813964

[B41] Scheltens P. De Strooper B. Kivipelto M. Holstege H. Chételat G. Teunissen C. E. (2021). Alzheimer's disease. Lancet 397 (10284), 1577–1590. 10.1016/s0140-6736(20)32205-4 33667416 PMC8354300

[B42] Sherawat K. Mehan S. (2023). Tanshinone-IIA mediated neuroprotection by modulating neuronal pathways. Naunyn Schmiedeb. Arch. Pharmacol. 396 (8), 1647–1667. 10.1007/s00210-023-02476-8 37010572

[B43] Subedi L. Gaire B. P. (2021). Tanshinone IIA: a phytochemical as a promising drug candidate for neurodegenerative diseases. Pharmacol. Res. 169, 105661. 10.1016/j.phrs.2021.105661 33971269

[B44] Thakur S. Dhapola R. Sarma P. Medhi B. Reddy D. H. (2023). Neuroinflammation in Alzheimer's disease: current progress in molecular signaling and therapeutics. Inflammation 46 (1), 1–17. 10.1007/s10753-022-01721-1 35986874

[B45] Walsh S. Merrick R. Milne R. Brayne C. (2021). Aducanumab for Alzheimer’s disease? Bmj 374, n1682. 10.1136/bmj.n1682 34226181 PMC8258645

[B46] Walsh S. Merrick R. Milne R. Nurock S. Richard E. Brayne C. (2024). Considering challenges for the new Alzheimer's drugs: clinical, population, and health system perspectives. Alzheimer's & Dementia 20, 6639–6646. 10.1002/alz.14108 PMC11497759 39105453

[B47] Wan C. Liu X. Q. Chen M. Ma H. H. Wu G. L. Qiao L. J. (2023). Tanshinone IIA ameliorates Aβ transendothelial transportation through SIRT1-mediated endoplasmic reticulum stress. J. Transl. Med. 21 (1), 34. 10.1186/s12967-023-03889-y 36670462 PMC9854034

[B48] Wang H. R. Li D. Weng H. C. Yue X. (2014). Study on pharmacoepidemiological characteristics of adverse reactions induced by sodium tanshinone ⅡASulfonate injection. China Pharm. 23 (14), 43–45.

[B49] Wen P. Y. Luo H. Zhou L. Song Z. Li W. W. Zhou J. (2014). Effects of tanshinone ⅡA on the expressions of caspase-3, Akt and NF-κB in the brains of rat models of Alzheimer's disease. Xibao Yu Fenzi Mianyixue Zazhi 30 (02), 155–159. 10.13423/j.cnki.cjcmi.007032 24491056

[B50] Xiang X. Xia S. Li S. Zeng Y. Wang L. Zhou Y. (2024a). Study on the role and mechanism of Tan IIA in Alzheimer's disease based on CREB-BDNF-TrkB pathway. Neurosci. Lett. 830, 137769. 10.1016/j.neulet.2024.137769 38616003

[B51] Xiang X. Y. Xia S. Y. Zhou Y. J. Li S. Wang L. X. Zeng Y. R. (2024b). Intervention effect of tanshinone ⅡA on streptozocin induced Alzheimer's disease in rats model and its mechanism. Guangxi Yixue 46 (11), 1698–1704. 10.11675/j.issn.0253-4304.2024.11.11

[B52] Yang L. X. Luo M. Li S. Y. (2024). Tanshinone IIA improves Alzheimer's disease *via* RNA nuclear-enriched abundant transcript 1/microRNA-291a-3p/member RAS oncogene family Rab22a axis. World J. psychiatry 14 (4), 563–581. 10.5498/wjp.v14.i4.563 38659601 PMC11036463

[B53] Zhai Z. Kong F. Zhu Z. Dai J. Cai J. Xie D. (2024). Effect and potential mechanism of immunotherapy on cognitive deficits in animal models of Alzheimer's disease: a systematic review and meta-analysis. Am. J. Geriatric Psychiatry 32 (5), 555–583. 10.1016/j.jagp.2023.11.011 38158285

[B54] Zhang D. P. Lu X. Y. He S. C. Li W. Y. Ao R. Leung F. C. (2020). Sodium tanshinone IIA sulfonate protects against Aβ-induced cell toxicity through regulating Aβ process. J. Cell Mol. Med. 24 (6), 3328–3335. 10.1111/jcmm.15006 31989795 PMC7131914

[B55] Zhang X. S. Ha S. Wang X. L. Shi Y. L. Duan S. S. Li Z. A. (2015). Tanshinone IIA protects dopaminergic neurons against 6-hydroxydopamine-induced neurotoxicity through miR-153/NF-E2-related factor 2/antioxidant response element signaling pathway. Neuroscience 303, 489–502. 10.1016/j.neuroscience.2015.06.030 26116522

[B56] Zhong C. Lin Z. Ke L. Shi P. Li S. Huang L. (2021). Recent research progress (2015–2021) and perspectives on the pharmacological effects and mechanisms of tanshinone IIA. Front. Pharmacol. 12, 778847. 10.3389/fphar.2021.778847 34819867 PMC8606659

