# Supplementary material for: Preclinical evidence and potential mechanisms of tanshinone ⅡA on cognitive function in animal models of Alzheimer’s disease: a systematic review and meta-analysis
Source: Front Pharmacol. 2025 Jul 11;16:1603861. doi: 10.3389/fphar.2025.1603861 (PMC12289687; doi:10.3389/fphar.2025.1603861)
Supplement: Supplementary file 1 [file DataSheet1.docx]

**Material S1. Search strategies in English databases**

**PubMed**

#1 " tanshinone " [Mesh]

#2 (((tanshinone[Title/Abstract]) OR ("tanshinone II A"[Title/Abstract])) OR ("tanshinone IIA"[Title/Abstract])) OR (TTE-50[Title/Abstract])

#3 #1OR#2

#4 "Alzheimer Disease" [MeSH Terms]

#5 ((((((((((((((("Alzheimer Disease"[Title/Abstract]) OR ("Alzheimer* Disease*"[Title/Abstract])) OR ("Alzheimer Syndrome"[Title/Abstract])) OR ("Alzheimer-Type Dementia"[Title/Abstract])) OR ("Alzheimer Type Dementia"[Title/Abstract])) OR ("Alzheimer Dementia*"[Title/Abstract])) OR ("Senile Dementia"[Title/Abstract])) OR ("Alzheimer Type Senile Dementia"[Title/Abstract])) OR ("Primary Senile Degenerative Dementia"[Title/Abstract])) OR ("Alzheimer Sclerosis"[Title/Abstract])) OR ("Presenile Dementia"[Title/Abstract])) OR ("Acute Confusional Senile Dementia"[Title/Abstract])) OR ("Early Onset Alzheimer Disease"[Title/Abstract])) OR ("Presenile Alzheimer Dementia"[Title/Abstract])) OR ("Late Onset Alzheimer Disease"[Title/Abstract])) OR ("Focal Onset Alzheimer's Disease"[Title/Abstract])

#6 #4OR#5

#7 "Animal Experimentation" [MeSH Terms]

#8 ((((("Animal Experiment*"[Title/Abstract]) OR ("in vivo"[Title/Abstract])) OR ("in vivo experiment*"[Title/Abstract])) OR (rats[Title/Abstract])) OR (mice[Title/Abstract])) OR ("animal models"[Title/Abstract])

#9 #7OR#8

#10 #3AND#6AND#9

**Embase**

#1 'tanshinone Ⅱa'/exp

#2 'tanshinone 2a':ab,ti OR 'tanshinone Ⅱ a':ab,ti OR 'tanshinone Ⅱa':ab,ti OR 'tte-50':ab,ti OR 'tanshinone':ab,ti

#3 #1OR#2

#4 'alzheimer disease'/exp

#5 'alzeimer disease':ab,ti OR 'alzheimer* disease*':ab,ti OR 'alzeimer syndrome':ab,ti OR 'alzheimer-type dementia':ab,ti OR 'alzheimer type dementia':ab,ti OR 'alzheimer* dementia':ab,ti OR 'senile dementia':ab,ti OR 'alzheimer type senile dementia':ab,ti OR 'primary senile degenerative dementia':ab,ti OR 'alzheimer sclerosis':ab,ti OR 'presenile dementia':ab,ti OR 'acute confusional senile dementia':ab,ti OR 'early onset alzheimer disease':ab,ti OR 'presenile alzheimer dementia':ab,ti OR 'late onset alzheimer disease':ab,ti OR 'focal onset alzheimer's disease':ab,ti OR 'familial alzheimer* disease*':ab,ti OR 'alzheimer fibrillary change':ab,ti OR 'alzheimer fibrillary lesion':ab,ti OR 'alzheimer neurofibrillary change':ab,ti OR 'alzheimer neuro* degeneration':ab,ti OR 'alzheimer perusini disease':ab,ti OR 'alzheimer sclerosis':ab,ti

#6 #4OR#5

#7 'animal experiment'/exp

#8 'animal experimentation':ab,ti OR 'animal physical conditioning':ab,ti OR 'animal studies':ab,ti OR 'animal study':ab,ti OR 'animal trial':ab,ti OR 'experiment, animal':ab,ti OR 'physical conditioning, animal':ab,ti OR 'animal experiment':ab,ti

#9 'in vivo':ab,ti OR 'in vivo experiment':ab,ti OR 'in vivo experiments':ab,ti OR 'rats':ab,ti OR 'mice':ab,ti OR 'animal models':ab,ti

#10 #7OR#8OR#9

#11 #3AND#6AND#10

**Web of Science**

#1 (((TS=(tanshinone)) OR TS=(tanshinone Ⅱ A)) OR TS=(tanshinone ⅡA)) OR TS=(TTE-50)

#2 (((((((((((((((TS=(Alzheimer* Disease*)) OR TS=(Alzheimer Syndrome)) OR TS=(Alzheimer-Type Dementia)) OR TS=(Alzheimer Type Dementia)) OR TS=(Alzheimer Dementia*)) OR TS=(Senile Dementia)) OR TS=(Alzheimer Type Senile Dementia)) OR TS=(Primary Senile Degenerative Dementia)) OR TS=(Alzheimer Sclerosis)) OR TS=(Presenile Dementia)) OR TS=(Acute Confusional Senile Dementia)) OR TS=(Early Onset Alzheimer Disease)) OR TS=(Presenile Alzheimer Dementia)) OR TS=(Late Onset Alzheimer Disease)) OR TS=(Focal Onset Alzheimer's Disease)) OR TS=(Familial Alzheimer* Disease*)

#3 ((((AB=(animal experiment*)) OR AB=(in vivo)) OR AB=(in vivo experiment*)) OR AB=(rats)) OR AB=(mice)

#4 #1AND#2AND#3

**Material S2. Search strategies in Chinese databases**

**CNKI**

(主题:丹参酮ⅡA +丹参酮 +TTE-50)AND(主题:阿尔茨海默病 +老年痴呆 +认知障碍 +认知)

**CBM**

("丹参酮ⅡA"[常用字段:智能] OR "丹参酮"[常用字段:智能] OR "TTE-50"[常用字段:智能]) AND ("阿尔茨海默病"[常用字段:智能] OR "老年痴呆"[常用字段:智能] OR "认知障碍"[常用字段:智能] OR "认知"[常用字段:智能])

**VIP**

题名或关键词 = 丹参酮ⅡA + 丹参酮 + TTE-50 AND 题名或关键词 = 阿尔茨海默病 +老年痴呆 +认知障碍 +认知

**Wanfang**

主题:(丹参酮ⅡA OR 丹参酮 OR TTE-50) and 主题:(阿尔茨海默病 OR 老年痴呆 OR 认知障碍 OR 认知)

**Table S1. Abstracted individual markers of brain injury and repair measured after Tan ⅡA therapy in animal models of** **Alzheimer's disease.**

| **Brain injury and repair markers*** | | | | | |
| --- | --- | --- | --- | --- | --- |
| **Pro-Inflammation** | **Pro-oxidative stress** | **Anti-oxidative stress** | **Pro- apoptosis** | **Anti- apoptosis** | **Pro- synaptic plasticity** |
| **Brain** | **Brain** | **Brain** | **Brain** | **Brain** | **Brain** |
| TNF-α | MDA | SOD | Caspase-3 | Bcl-2/Bax | PSD-95 |
| IL-1β | ROS | GSH-Px |  |  | BDNF |
| IL-6 |  |  |  |  |  |

Bcl-2/Bax: B-cell lymphoma-2/Bcl-2-associated X protein; BDNF: brain-derived neurotrophic factor; GSH-Px: glutathione peroxidase; IL-1β: interleukin-1beta; IL-6: interleukin-6; MDA: malondialdehyde; PSD-95: postsynaptic density-95; ROS: reactive oxygen species; SOD: superoxide dismutase; TNF-α: tumor necrosis factor-alpha

*Most markers measured protein levels unless otherwise specified.

**Table S2.** **Subgroup analyses** **of hippocampal Aβ immunohistochemistry staining area.**

| Subgroup | SMD | LL | HL | I^2^ | Z | P |
| --- | --- | --- | --- | --- | --- | --- |
| Animal species | | | | | | |
| Mice | -3.27 | -5.07 | -1.48 | 50% | 3.57 | ＜0.01 |
| Rats | 0.90 | -0.44 | 2.24 | - | 1.32 | 0.19 (＞0.05) |
| Model | | | | | | |
| Aβ_1-42_ | 0.90 | -0.44 | 2.24 | - | 1.32 | 0.19 (＞0.05) |
| Transgenic AD | -2.70 | -4.90 | -0.51 | 57% | 2.41 | 0.02 (＜0.05) |
| STZ | -4.73 | -7.34 | -2.13 | - | 3.56 | ＜0.01 |
| Dose | | | | | | |
| ≤40mg/Kg | -1.40 | -4.31 | 1.51 | 87% | 0.94 | 0.35 (＞0.05) |
| ＞40mg/Kg | -4.73 | -7.34 | -2.13 | - | 3.56 | ＜0.01 |
| Treatment duration | | | | | | |
| ≤30 days | -2.43 | -6.17 | 1.32 | 91% | 1.27 | 0.20 (＞0.05) |
| ＞30 days | -1.52 | -3.69 | 0.65 | - | 1.37 | 0.17 (＞0.05) |

**Table S3. Subgroup analyses of TNF-α.**

| Subgroup | SMD | LL | HL | I^2^ | Z | P |
| --- | --- | --- | --- | --- | --- | --- |
| Animal species | | | | | | |
| Mice | -1.80 | -2.57 | -1.03 | 0% | 4.58 | ＜0.01 |
| Rats | -5.20 | -7.56 | -2.83 | - | 4.31 | ＜0.01 |
| Model | | | | | | |
| Aβ_1-42_ | -3.03 | -5.36 | -0.71 | - | 2.56 | 0.01 |
| Transgenic AD | -1.90 | -3.24 | -0.57 | - | 2.80 | ＜0.01 |
| STZ | -5.20 | -7.56 | -2.83 | - | 4.31 | ＜0.01 |
| LPS | -1.49 | -2.53 | -0.46 | - | 2.84 | ＜0.01 |
| Administration route | | | | | | |
| Intraperitoneal injection | -1.80 | -2.57 | -1.03 | 0% | 4.58 | ＜0.01 |
| Oral gavage | -5.20 | -7.56 | -2.83 | - | 4.31 | ＜0.01 |
| Dose | | | | | | |
| ≤40mg/Kg | -1.80 | -2.57 | -1.03 | 0% | 4.58 | ＜0.01 |
| ＞40mg/Kg | -5.20 | -7.56 | -2.83 | - | 4.31 | ＜0.01 |
| Treatment duration | | | | | | |
| ≤30 days | -3.20 | -5.13 | -1.26 | 65% | 3.24 | ＜0.01 |
| ＞30 days | -1.49 | -2.53 | -0.46 | - | 2.84 | ＜0.01 |

**Table S4. Subgroup analyses of IL-1β.**

| Subgroup | SMD | LL | HL | I^2^ | Z | P |
| --- | --- | --- | --- | --- | --- | --- |
| Animal species | | | | | | |
| Mice | -1.94 | -3.02 | -0.85 | 0% | 3.49 | ＜0.01 |
| Rats | -6.16 | -13.11 | 0.79 | 81% | 1.74 | 0.08 (＞0.05) |
| Model | | | | | | |
| Aβ_1-42_ | -5.79 | -13.66 | 2.07 | 84% | 1.44 | 0.15 (＞0.05) |
| Transgenic AD | -1.79 | -3.10 | -0.49 | - | 2.69 | ＜0.01 |
| STZ | -3.16 | -4.85 | -1.46 | - | 3.65 | ＜0.01 |
| Administration route | | | | | | |
| Intraperitoneal injection | -3.21 | -5.87 | -0.55 | 74% | 2.36 | 0.02 (＜0.05) |
| Oral gavage | -3.16 | -4.85 | -1.46 | - | 3.65 | ＜0.01 |
| Dose | | | | | | |
| ≤40mg/Kg | -3.21 | -5.87 | -0.55 | 74% | 2.36 | 0.02 (＜0.05) |
| ＞40mg/Kg | -3.16 | -4.85 | -1.46 | - | 3.65 | ＜0.01 |

**Table S5. Subgroup analyses of IL-6.**

| Subgroup | SMD | LL | HL | I^2^ | Z | P |
| --- | --- | --- | --- | --- | --- | --- |
| Animal species | | | | | | |
| Mice | -2.16 | -3.65 | -0.67 | 62% | 2.84 | ＜0.01 |
| Rats | -1.69 | -3.00 | -0.37 | - | 2.51 | 0.01 |
| Model | | | | | | |
| Aβ_1-42_ | -3.35 | -5.84 | -0.87 | - | 2.64 | ＜0.01 |
| Transgenic AD | -2.83 | -4.42 | -1.23 | - | 3.48 | ＜0.01 |
| STZ | -1.69 | -3.00 | -0.37 | - | 2.51 | 0.01 |
| LPS | -1.06 | -2.05 | -0.08 | - | 2.13 | 0.03 (＜0.05) |
| Administration route | | | | | | |
| Intraperitoneal injection | -2.16 | -3.65 | -0.67 | 62% | 2.84 | ＜0.01 |
| Oral gavage | -1.69 | -3.00 | -0.37 | - | 2.51 | 0.01 |
| Dose | | | | | | |
| ≤40mg/Kg | -2.16 | -3.65 | -0.67 | 62% | 2.84 | ＜0.01 |
| ＞40mg/Kg | -1.69 | -3.00 | -0.37 | - | 2.51 | 0.01 |
| Treatment duration | | | | | | |
| ≤30 days | -2.32 | -3.26 | -1.38 | 0% | 4.84 | ＜0.01 |
| ＞30 days | -1.06 | -2.05 | -0.08 | - | 2.13 | 0.03 (＜0.05) |

**Table S6. Subgroup analyses of Caspase-3.**

| Subgroup | SMD | LL | HL | I^2^ | Z | P |
| --- | --- | --- | --- | --- | --- | --- |
| Animal species | | | | | | |
| Mice | -2.35 | -3.10 | -1.59 | 28% | 6.09 | ＜0.01 |
| Rats | -3.29 | -5.59 | -1.00 | 53% | 2.82 | ＜0.01 |
| Model | | | | | | |
| Aβ_1-42_ | -3.29 | -5.59 | -1.00 | 53% | 2.82 | ＜0.01 |
| Transgenic AD | -2.43 | -3.47 | -1.38 | 46% | 4.54 | ＜0.01 |
| STZ | -2.35 | -3.55 | -1.15 | - | 3.83 | ＜0.01 |
| Administration route | | | | | | |
| Intraperitoneal injection | -2.62 | -3.60 | -1.64 | 0% | 5.23 | ＜0.01 |
| Oral gavage | -2.54 | -3.60 | -1.48 | 48% | 4.69 | ＜0.01 |
| Dose | | | | | | |
| ≤40mg/Kg | -2.35 | -3.17 | -1.53 | 28% | 2.61 | ＜0.01 |
| ＞40mg/Kg | -3.21 | -5.42 | -1.00 | 60% | 2.85 | ＜0.01 |
| Treatment duration | | | | | | |
| ≤30 days | -2.43 | -3.38 | -1.48 | 46% | 5.02 | ＜0.01 |
| ＞30 days | -2.88 | -4.03 | -1.73 | 0% | 4.90 | ＜0.01 |

**Table S7. Subgroup analyses of Bcl-2/Bax ratio.**

| Subgroup | SMD | LL | HL | I^2^ | Z | P |
| --- | --- | --- | --- | --- | --- | --- |
| Model | | | | | | |
| Aβ_1-42_ | 8.73 | 3.18 | 14.28 | - | 3.08 | ＜0.01 |
| Transgenic AD | 4.84 | 1.23 | 8.44 | 91% | 2.63 | ＜0.01 |
| STZ | 3.62 | 2.10 | 5.13 | - | 4.68 | ＜0.01 |
| Administration route | | | | | | |
| Intraperitoneal injection | 4.00 | 2.38 | 5.61 | 36% | 4.86 | ＜0.01 |
| Oral gavage | 7.04 | 1.31 | 12.76 | 93% | 2.41 | 0.02 (＜0.05) |
| Dose | | | | | | |
| ≤40mg/Kg | 5.54 | 2.15 | 8.94 | 89% | 3.20 | ＜0.01 |
| ＞40mg/Kg | 3.62 | 2.10 | 5.13 | - | 4.68 | ＜0.01 |
| Treatment duration | | | | | | |
| ≤30 days | 3.36 | 1.80 | 4.92 | 63% | 4.21 | ＜0.01 |
| ＞30 days | 19.75 | -15.69 | 55.19 | 97% | 1.09 | 0.27 (＞0.05) |

**Table S8. Subgroup analyses of BDNF.**

| Subgroup | SMD | LL | HL | I^2^ | Z | P |
| --- | --- | --- | --- | --- | --- | --- |
| Animal species | | | | | | |
| Mice | 3.39 | 1.02 | 5.77 | 88% | 2.80 | ＜0.01 |
| Rats | 1.81 | 0.47 | 3.15 | - | 2.64 | ＜0.01 |
| Model | | | | | | |
| LPS | 1.33 | 0.32 | 2.34 | - | 2.58 | 0.01 |
| Transgenic AD | 4.69 | 0.63 | 8.75 | 91% | 2.26 | 0.02 (＜0.05) |
| STZ | 1.81 | 0.47 | 3.15 | - | 2.64 | ＜0.01 |
| Administration route | | | | | | |
| Intraperitoneal injection | 1.48 | 0.68 | 2.27 | 0% | 3.64 | ＜0.01 |
| Oral gavage | 4.72 | 0.66 | 8.79 | 90% | 2.28 | 0.02 (＜0.05) |
| Dose | | | | | | |
| ≤40mg/Kg | 4.54 | 0.45 | 8.62 | 92% | 2.18 | 0.03 (＜0.05) |
| ＞40mg/Kg | 1.76 | 0.83 | 2.69 | 0% | 3.71 | ＜0.01 |
| Treatment duration | | | | | | |
| ≤30 days | 1.76 | 0.83 | 2.69 | 0% | 3.71 | ＜0.01 |
| ＞30 days | 4.54 | 0.45 | 8.62 | 92% | 2.18 | 0.03 (＜0.05) |

**Table S9. Meta-regression analysis for escape latency in Morris water maze.**

| Outcomes | Characteristics (covariates)  (*n* = 14) | Coefficient | 95% CI | *p* value |
| --- | --- | --- | --- | --- |
| Escape latency | Study quality | 0.06 | [-1.45, 1.56] | 0.93 |
|  | Animal species | 0.41 | [-1.12, 1.93] | 0.57 |
|  | Model type | 0.16 | [-0.41, 0.72] | 0.56 |
|  | Administration route | -0.02 | [-1.51, 1.47] | 0.98 |
|  | Dose | 1.00 | [-0.36, 2.36] | 0.14 |
|  | Treatment duration | -2.11 | [-3.43, -0.79] | **＜0.05** |

**Table S10. Meta-regression analysis for** **time spent in the target quadrant in Morris water maze.**

| Outcomes | Characteristics (covariates)  (*n* = 10) | Coefficient | 95% CI | *p* value |
| --- | --- | --- | --- | --- |
| Time spent in the target quadrant | Study quality | -0.33 | [-1.63, 0.98] | 0.58 |
|  | Animal species | -0.34 | [-1.82, 1.13] | 0.60 |
|  | Model type | 0.05 | [-0.47, 0.58] | 0.82 |
|  | Administration route | -0.10 | [-1.31, 1.11] | 0.85 |
|  | Dose | -0.40 | [-1.56, 0.76] | 0.45 |
|  | Treatment duration | -0.48 | [-1.93, 0.97] | 0.47 |

**Table S11. Meta-regression analysis for SOD.**

| Outcomes | Characteristics (covariates)  (*n* = 7) | Coefficient | 95% CI | *p* value |
| --- | --- | --- | --- | --- |
| SOD | Study quality | -0.73 | [-3.61, 2.15] | 0.54 |
|  | Animal species | 0.04 | [-2.81, 2.89] | 0.97 |
|  | Model type | -0.35 | [-1.94, 1.24] | 0.59 |
|  | Administration route | -0.10 | [-2.51, 2.71] | 0.93 |
|  | Dose | -1.23 | [-3.29, 0.84] | 0.19 |
|  | Treatment duration | -0.90 | [-1.79, 3.59] | 0.43 |

**Table S12. Meta-regression analysis for MDA.**

| Outcomes | Characteristics (covariates)  (*n* = 7) | Coefficient | 95% CI | *p* value |
| --- | --- | --- | --- | --- |
| MDA | Study quality | 1.54 | [-1.17, 4.26] | 0.20 |
|  | Animal species | -0.94 | [-3.66, 1.79] | 0.42 |
|  | Model type | -0.95 | [-2.37, 0.46] | 0.14 |
|  | Administration route | 1.25 | [-1.00, 3.50] | 0.21 |
|  | Dose | -0.18 | [-2.81, 2.46] | 0.87 |
|  | Treatment duration | 1.22 | [-1.12, 3.56] | 0.24 |

# Figure S1. Reporting quality assessment of included studies

# Figure S2. Effect of tanshinone ⅡA on SOD and GSH-Px
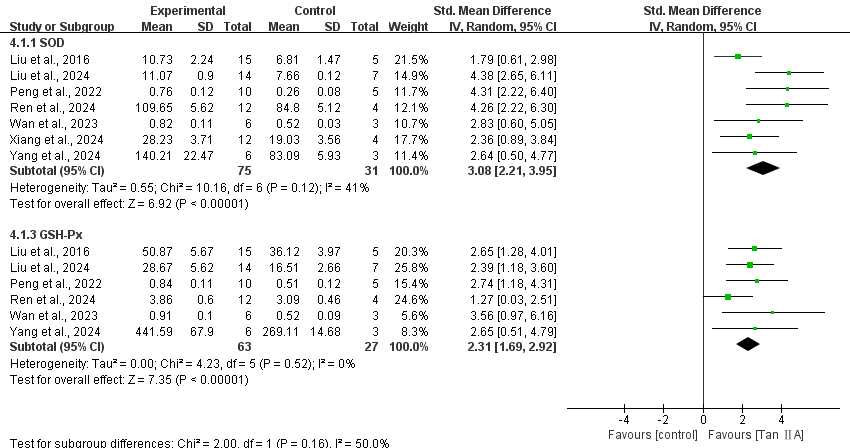
.

**Figure S3. Effect of tanshinone ⅡA on MDA and ROS.**


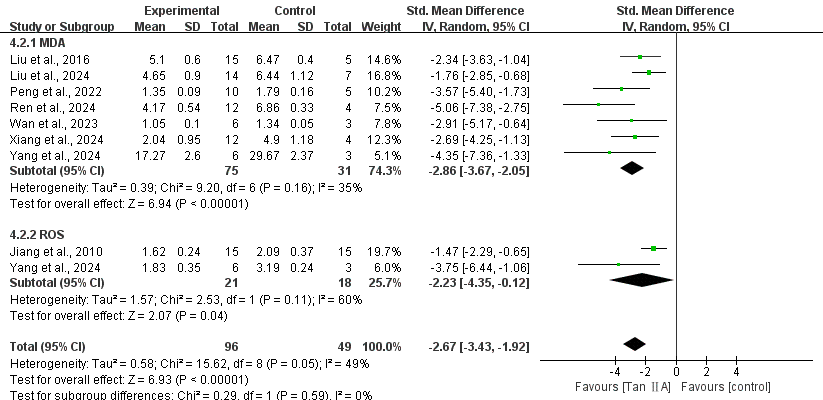


**Figure S4. Effect of tanshinone ⅡA on Caspase-3 and Bcl-2/Bax.**


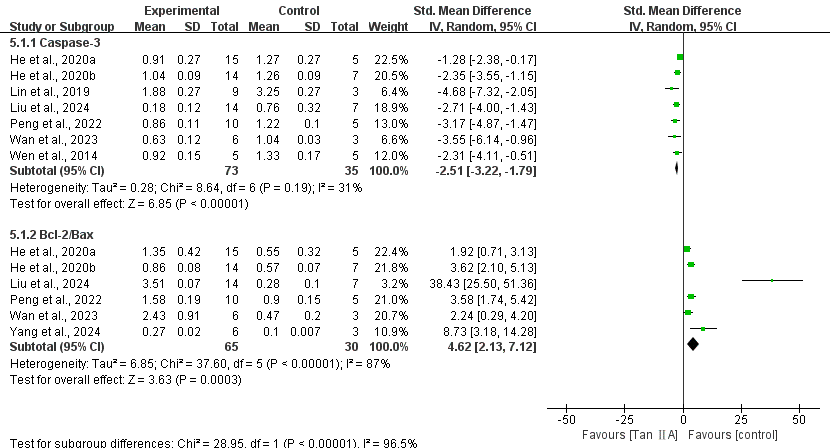


**Figure S5. Effect of tanshinone ⅡA on PSD-95 and BDNF.**


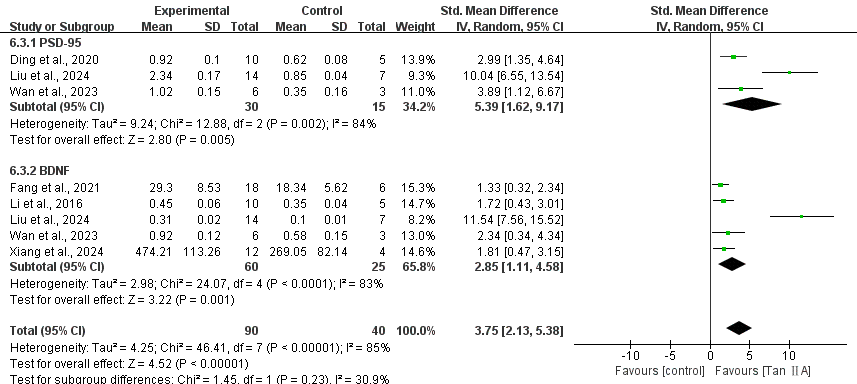


**Figure S6. Filled funnel plot of escape latency,** **time spent in the target quadrant, SOD and MDA.**


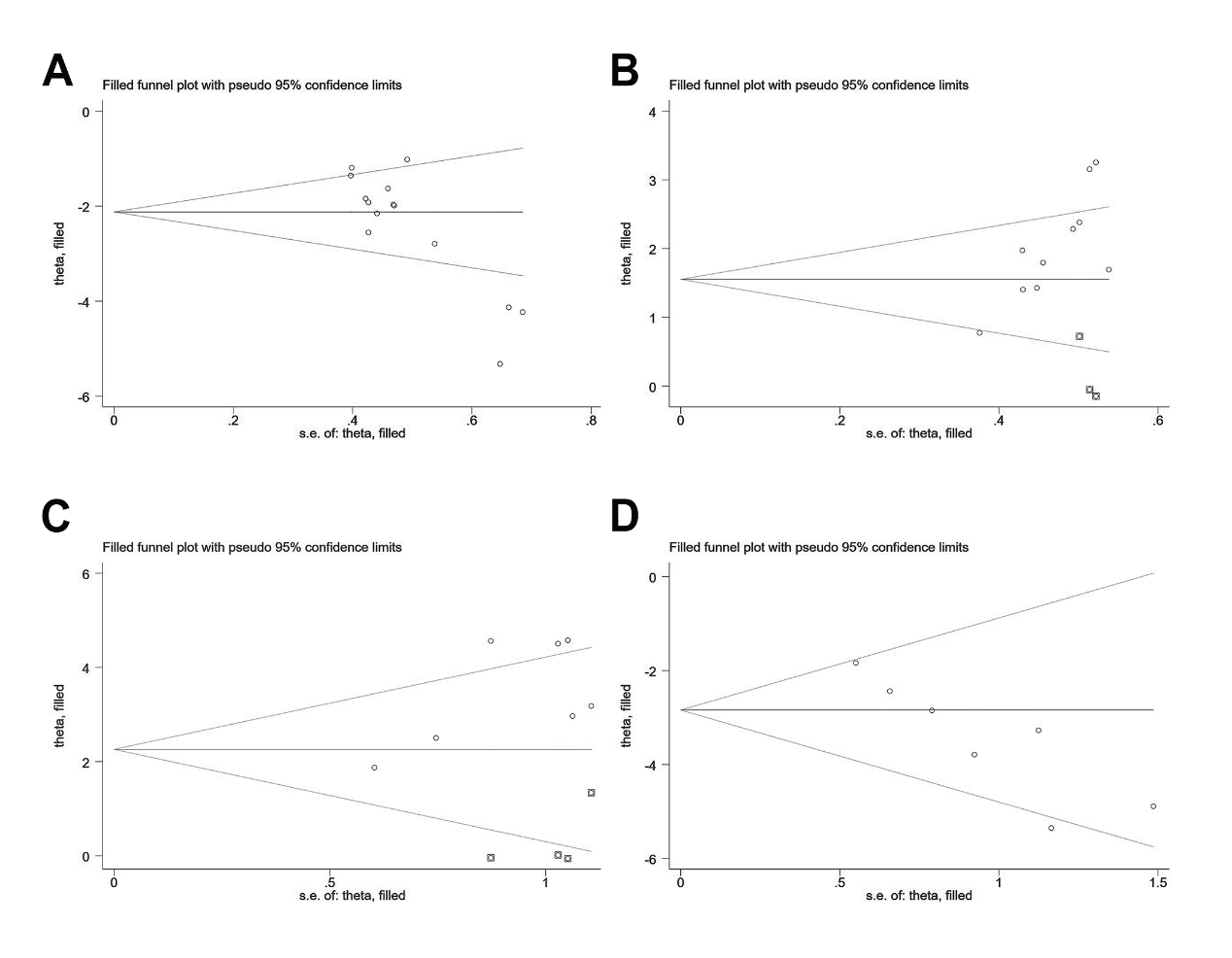


(A) escape latency; (B) time spent in the target quadrant; (C) SOD; (D) MDA.
